# Mec1-Rad53 Signaling Regulates DNA Damage-Induced Autophagy and Pathogenicity in *Candida albicans*

**DOI:** 10.3390/jof9121181

**Published:** 2023-12-09

**Authors:** Jiawen Du, Yixuan Dong, Wenjie Zuo, Ying Deng, Hangqi Zhu, Qilin Yu, Mingchun Li

**Affiliations:** Key Laboratory of Molecular Microbiology and Technology, Ministry of Education, Department of Microbiology, College of Life Sciences, Nankai University, 94 Weijin Road, Nankai District, Tianjin 300071, China; dujiawen0919@163.com (J.D.); dyx178606206862021@163.com (Y.D.); zuowenjiezz@163.com (W.Z.); dy19881702071@163.com (Y.D.); zhuhangqi0922@163.com (H.Z.); yuqilin@mail.nankai.edu.cn (Q.Y.)

**Keywords:** DNA damage-induced autophagy, Mec1-Rad53 signaling, Atg1, Atg13, autophagosome biogenesis, *Candida albicans*

## Abstract

DNA damage activates the DNA damage response and autophagy in *C. albicans*; however, the relationship between the DNA damage response and DNA damage-induced autophagy in *C. albicans* remains unclear. Mec1-Rad53 signaling is a critical pathway in the DNA damage response, but its role in DNA damage-induced autophagy and pathogenicity in *C. albicans* remains to be further explored. In this study, we compared the function of autophagy-related (Atg) proteins in DNA damage-induced autophagy and traditional macroautophagy and explored the role of Mec1-Rad53 signaling in regulating DNA damage-induced autophagy and pathogenicity. We found that core Atg proteins are required for these two types of autophagy, while the function of Atg17 is slightly different. Our results showed that Mec1-Rad53 signaling specifically regulates DNA damage-induced autophagy but has no effect on macroautophagy. The recruitment of Atg1 and Atg13 to phagophore assembly sites (PAS) was significantly inhibited in the *mec1*Δ/Δ and *rad53*Δ/Δ strains. The formation of autophagic bodies was obviously affected in the *mec1*Δ/Δ and *rad53*Δ/Δ strains. We found that DNA damage does not induce mitophagy and ER autophagy. We also identified two regulators of DNA damage-induced autophagy, Psp2 and Dcp2, which regulate DNA damage-induced autophagy by affecting the protein levels of Atg1, Atg13, Mec1, and Rad53. The deletion of Mec1 or Rad53 significantly reduces the ability of *C. albicans* to systematically infect mice and colonize the kidneys, and it makes *C. albicans* more susceptible to being killed by macrophages.

## 1. Introduction

Living organisms are constantly facing internal and external threats that cause genomic instability; internal threats, including reactive oxygen species (ROS) and DNA replication; and external threats, including chemical and physical genotoxic DNA damage agents [1,2]. To resist both internal and external DNA damage stresses, living organisms have evolved conserved DNA damage response pathways [3]. Ataxia-telangiectasia mutated (ATM) and ATM- and Rad3-related (ATR) mediated DNA damage response (DDR) is essential for maintaining genomic stability and is evolutionarily conserved from yeasts to humans [4,5]. In humans, ATR and ATM kinases are members of the phosphatidylinositide-3-kinase-like-kinase (PI3KK) family, and Chk1 and Chk2 are their downstream effector kinases [6]. The ATR/Chk1 pathway usually responds to single-stranded DNA or bulky DNA lesions, while the ATM/Chk2 pathway usually responds to double strand DNA breaks [7,8]. In *Saccharomyces cerevisiae*, the DNA damage response is mediated by Mec1 (a homolog of human ATR) and Tel1 (a homolog of human ATM) [9]. Rad53 (a homolog of human Chk2) and Chk1 are downstream effector kinases, and Mec1 activates them via phosphorylation of their Ser/Thr phosphorylation (SQ/TQ) sites [10].

Autophagy is an evolutionarily conserved cellular process that can degrade various components of cells to produce biological macromolecules for cell reuse [11]. According to the different substrates targeted, autophagy can be divided into non-selective autophagy (macroautophagy) and selective autophagy [12]. Autophagy is activated when cells feel various environmental stresses, saving cells from critical environments [12]. The bilayer autophagosome is formed gradually during autophagy and is responsible for transporting intracellular components to lysosomes or vacuoles for degradation [13,14]. More than 40 autophagy-related (Atg) proteins have been identified as required for autophagy [15]. Autophagy is regulated by core autophagy-related (Atg) proteins or complexes: (1) the initiation complex of Atg1, Atg13, Atg17, Atg29, Atg31 [16]; (2) ubiquitin-like protein conjugation systems with Atg12-Atg5-Atg16 and Atg8 [17]; (3) the Atg2-Atg18 complex [18]; (4) the transmembrane protein, Atg9 [19]; and (5) the PtdIns3K (phosphatidylinositol 3-kinase) complex with Atg6, Atg14, Vps15, and Vps34 [20]. The nutrient sensing kinase target of rapamycin complex 1 (TORC1) is the primary regulator in autophagy, which affects the formation of the Atg1 complex (Atg1-Atg13-Atg17-Atg29-Atg31) by influencing the phosphorylation of Atg13, thus regulating the initiation of autophagy [16].

In mammalian cells and yeast, autophagy can be induced in response to DNA-damaging agents, such as methyl methanesulfonate (MMS), hydroxyurea (HU), and zeocin [21,22]. Autophagy is involved in the resolution of DNA lesions and plays complex roles in the context of DNA damage and repair. In tumor cells, autophagy has been proved to be involved in DNA repair by clearing damaged DNA induced by internal and/or external factors [23]. Autophagy can also facilitate cell death by promoting degradation of DNA damage repair proteins [24,25]. DNA repair proteins have been shown to be involved in the activation of autophagy caused by genotoxic stresses [26,27,28]. In mammalian cells, DDR signaling can induce DNA damage-induced autophagy by inhibiting mTORC1 activity through various pathways, such as ATM, p53, AMP-activated protein kinase (AMPK) [29,30,31]. ATR/Chk1 signaling is also involved in the activation of DNA damage-induced autophagy [21]. In *S. cerevisiae*, it has been shown that DDR plays a critical role in the regulation of DNA damage-induced autophagy [22]. However, whether DDR regulates autophagy in *C. albicans* and the underlying mechanisms remain unclear. Many studies have shown that DDR plays an important role in the virulence of pathogens. In *Cryptococcus neoformans*, the perturbation of both *CHK1* and *RAD53* attenuated the virulence [1]. In *Aspergillus fumigatus*, the absence of *RTT109* leads to hypersensitivity to genotoxic agents, which causes a reduction in virulence [32]. In *Acinetobacter baumannii*, RecA is involved in the regulation of DNA repair responses, and the deletion of DDR-related protein RecA results in attenuated virulence [33]. In *Fusarium graminearum*, the strains lacking DDR-related gene *RAD50* showed reduced virulence [34]. In *C. albicans*, DDR has a dual regulatory effect on virulence [35]. The deletion of *RTT109* weakens the tolerance to DNA damage stressing agents and attenuates the virulence in *C. albicans* [36]. The absence of *RAD52* also reduces virulence in *C. albicans* [37]. The expression of *RAD6* and *DDR48* are regulated by *RFX2*, which is a DNA damage responsive gene [38]. The strains lacking *RFX2* exhibits attenuated virulence in *C. albicans* [38]. The DDR-related genes also play negative roles in virulence of *C. albicans*, such as *PPH3* and *PSY2* [39]. However, whether Mec1-Rad53 signaling participates in the regulation of virulence in *C. albicans* and whether it has a positive or negative role are still unclear.

In this study, we identified the regulatory role of Mec1-Rad53 signaling on DNA damage-induced autophagy in *C. albicans*. We found that the deletion of Mec1 or Rad53 significantly inhibits DNA damage-induced autophagy but has no effect on nitrogen starvation-induced autophagy. The strains lacking Mec1 or Rad53 exhibit less Atg1 and Atg13 puncta and autophagic bodies. The RNA binding proteins, Psp2 and Dcp2, are involved in the regulation of DNA damage-induced autophagy in *C. albicans*. We also found that Mec1-Rad53 signaling plays a positive role in the virulence of *C. albicans*. These findings advance our understanding of the interaction between the DDR pathway and autophagy in *C. albicans* as well as the regulation of pathogenic virulence through the DDR pathway.

## 2. Materials and Methods

### 2.1. Strains and Growth Conditions

The *C. albicans* strains used in this study were derived from wild-type (WT) BWP17 and are listed in Table S1. YPD medium (1% yeast extract, 2% peptone, 2% glucose) with or without 80 μg/mL uridine (Sangon Biotech, Shanghai, China) was used for the culture of strains. SC (synthetic complete) medium (0.67% yeast nitrogen base without amino acids, 2% glucose, and 0.2% complete amino acid mixture) with or without 80 μg/mL uridine was used for screening transformants. Cells were cultured in YPD medium to logarithmic phase, and autophagy was induced by adding MMS or being transferred to MM-N medium (1.04% (*m*/*v*) MgSO_4_·7H_2_O, 3.04% (*m*/*v*) KH_2_PO_4_, 1.04% (*m*/*v*) KCl, 0.1% (*v*/*v*) 1000 × trace element solution, 0.1% (*v*/*v*) 1000 × vitamin solution).

### 2.2. The Construction of Plasmids and Strains

The plasmids and primers used in this study are listed in Tables S1 and S2. pGFP-Atg8 contains a pAU34M backbone and the *ACT1* promoter. The linearized pGFP-Atg8 was transformed into *atg*Δ/Δ strains to obtain the *atg*Δ/Δ-GFP-Atg8 strains. pGFP-Atg1 and pGFP-Atg13 were modified on the basis of pGFP-Atg8 and also contained the *ACT1* promoter. To obtain the plasmids containing *ATG13^WT^*, *ATG13^1-268aa^*^Δ/Δ^, *ATG13^269-738aa^*^Δ/Δ^, *ATG13^461-640aa^*^Δ/Δ^, *ATG13^461-474aa^*^Δ/Δ^, PCR fragments were generated with fusion PCR and cloned into pDDB78. PCR-mediated homologous integration was used to construct these mutant strains. The method of gene knockout is described below. The *ARG4* cassette was amplified from the plasmid pRS-ArgΔSpeI and transformed into BWP17 to obtain the heterozygous mutant. The *URA3* cassette amplified from the plasmid pDDB57 was transformed into a heterozygous mutant to obtain a homozygous mutant. 5-Fluoroorotic acid selection was used to re-delete *URA3*. The fragment containing HA tags was amplified from the plasmid pHA-*URA3* and was transformed into WT and mutant strains, and the homologous arms were designed in the primers. The related strains were obtained by transforming linearized-related plasmids into WT and mutant strains.

### 2.3. Immunoblotting

The strains were cultured and induced as previously described. After being collected by centrifugation, the cells were lysed via vortexing. Radio Immunoprecipitation Assay (RIPA) lysis buffer (50 mM Tris (pH 7.4), 150 mM NaCl, 0.5% sodium deoxycholate, 1% NP-40, 1 mM EDTA) was used to lyse cells. The complete protease inhibitors (Roche, Basel, Switzerland) were added into the lysis buffer to prevent protein degradation. Protein concentration was measured by the BCA protein concentration assay (Solarbio, Beijing, China). The molecular weight marker we used in this study was Blue Plus II (TransGen, Beijing, China). Followed by standard sodium dodecyl sulphate polyacrylamide gel electrophoresis SDS-PAGE, separated proteins were transferred onto PVDF membranes. After being blocked by 5% skim milk, the membranes were probed with GFP monoclonal antibody (1:3000; MBL, Nagoya, Japan), α-Tubulin monoclonal antibody (1:3000; MBL), and HA monoclonal antibody (1:3000; Sigma, St. Louis, MI, USA). HRP-conjugated goat anti-mouse IgG (1:5000; BioRad, Hercules, CA, USA) was used as the secondary antibody.

### 2.4. Fluorescence Microscopy

The control and treated cells were collected and suspended in phosphate-buffered saline; after being stained with 4% FM4-64 (50 μg/mL, prepared in dimethyl sulfoxide (DMSO), Sigma, St. Louis, MI, USA), the cells were observed with a fluorescence microscope (Olympus, Tokyo, Japan). Cells containing GFP-Atg8 were observed using green fluorescence filters, whereas cells stained with FM4-64 were observed using TRITC/Texas Red filters. The number of cells was counted, and at least 300 cells were counted.

### 2.5. Transmission Electron Microscopy (TEM)

Control and treated cells were collected via centrifugation and washed twice with phosphate-buffered saline. The cells were fixed in 2% glutaraldehyde solution at 4 °C, dehydrated with graded ethanol, and cut into copper grids. Ethanolic uranyl acetate and lead citrate were used to stain the cells. The autophagic bodies in vacuoles were observed with transmission electron microscopy (TEM; Tecnai G2 F-20, FEI, Hillsboro, OR, USA). The number of autophagic bodies was counted, and at least 100 cells were observed.

### 2.6. Virulence Assays

The cells activated overnight were cultured to logarithmic phase. After being centrifuged, the cells were suspended in normal saline. After its density was adjusted to 8 × 10^6^ cells/mL, 100 μL of cell suspension was injected into the tail veins of mice. ICR female (4–5 weeks old) was used to determine the virulence of *C. albicans*. The survival of each group was monitored over 22 days. The mice were sacrificed after 6 days to measure the fungal burdens in kidneys of mice. After grinding the kidneys, the grinding solution was spread onto the YPD plates according to the appropriate dilution, and the number of colonies on the YPD plates was counted. Data were analyzed using GraphPad Prism (Version 8.3, GraphPaD, San Diego, CA, USA). For histopathological analysis, after being fixed overnight in 4% formaldehyde, the kidneys were embedded in paraffin, then cut and stained with haematoxylin and eosin (H and E). A light microscope (BX51, Olympus, Tokyo, Japan) were used to observe the stained sections. All murine experiments were approved by the Institutional Animal Care and Use Committee of Nankai University.

### 2.7. Assay of Interaction between C. albicans and Macrophages

The macrophage we used was RAW 264.7, which was derived from mice. Macrophages were cultured in 24-well plates in the density of 2 × 10^3^ cells/mL in Dulbecco’s modification of Eagle’s medium Dulbecco (DMEM), containing 10% FBS, penicillin, and streptomycin at 37 °C, and they were passed for two consecutive generations to ensure cell viability. *C. albicans* was cultured to the logarithmic phase. After being collected, the strains were suspended, and the OD_600_ of the strains was adjusted to 0.2. The macrophages and *C. albicans* were co-cultured at 37 °C for 1 h 40 min and were fixed with 4% formaldehyde solution. The samples were dehydrated by 30%, 50%, 70%, 80%, 95%, and 100% ethanol solution for 30 min each and frozen dry for 5 to 6 h. The scanning electron microscope (Quanta SEM, FEI, Hillsboro, OR, USA) was used to observe the samples.

### 2.8. Statistical Analysis

Each experiment was performed three times. Data are presented as mean ± standard error of the mean (SEM), *n* = 3. The statistical significance of the data was calculated with Student’s two-tailed *t*-tests. The statistical analyses were performed with GraphPad Prism (version 8.3, GraphPad Software, San Diego, CA, USA). Statistical probability of * *p* < 0.05, ** *p* < 0.01, and *** *p* < 0.001 were considered statistically significant.

## 3. Results

### 3.1. Comparison of the Role of Atg Proteins in DNA Damage-Induced Autophagy and Macroautophagy

**Figure 1:**
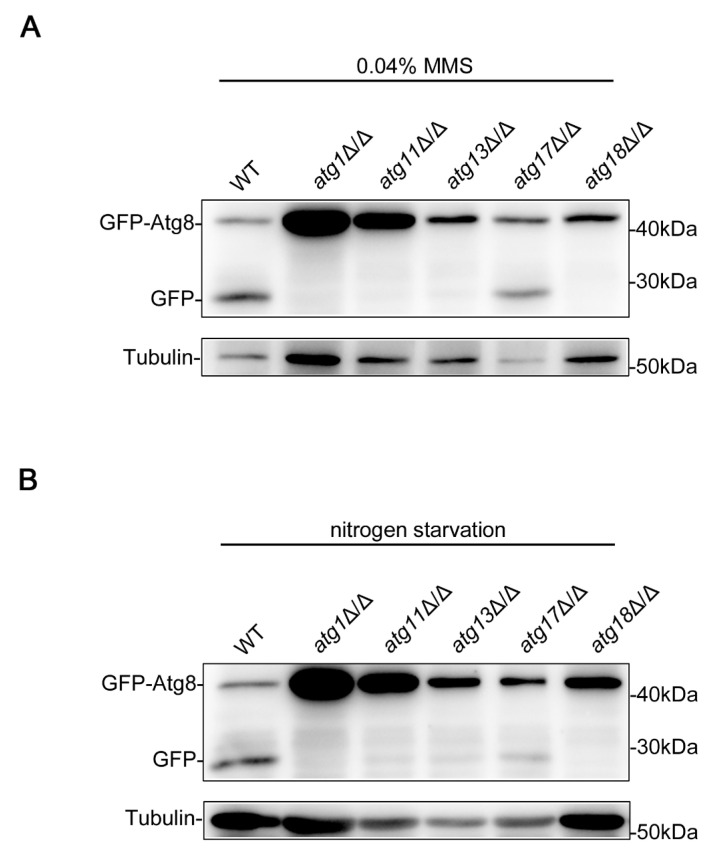
Function comparison of Atg proteins in DNA damage-induced autophagy and macroautophagy. Immunoblotting assay for GFP-Atg8 cleavage under an MMS treatment (**A**) and nitrogen starvation (**B**). GFP-Atg8 was expressed in the WT, *atg1*Δ/Δ, *atg11*Δ/Δ, *atg13*Δ/Δ, *atg17*Δ/Δ, and *atg18*Δ/Δ strains, and these strains were treated with MMS for 4 h or subjected to nitrogen starvation for 2 h. Immunoblots were performed using the GFP antibody. The levels of Tubulin served as loading controls.

DNA damage has been shown to induce autophagy in mammalian cells and yeast [21,22], and in our previous work, we also found that DNA damage can induce autophagy in *C. albicans* [40]. The traditional and best-studied autophagy is macroautophagy, also known as non-selective autophagy [41], which is usually activated under nitrogen starvation conditions [42]. Atg proteins play an essential role in autophagy, regulating autophagy at various stages [16,17,18,19,20]. In *S. cerevisiae*, it has been proven that Atg proteins play different roles in glucose starvation-induced autophagy and nitrogen starvation-induced autophagy (macroautophagy) [43], indicating that different types of autophagy may not require exactly the same Atg proteins. To determine whether the Atg proteins required for DNA damage-induced autophagy and nitrogen starvation-induced autophagy are different, we examined the autophagy of strains lacking Atg proteins under an MMS treatment and nitrogen starvation, and the degree of autophagy was represented by the degree of transition from GFP-Atg8 to GFP [44,45]. Atg8 is subject to vacuolar proteolysis, while GFP is more resistant to proteolysis during autophagy; therefore, the accumulation of free GFP is a measure of autophagic function. The results showed that the *atg1*Δ/Δ, *atg11*Δ/Δ, *atg13*Δ/Δ, and *atg18*Δ/Δ strains have almost no GFP-Atg8 to GFP conversion under an MMS treatment and nitrogen starvation (Figure 1), suggesting that these Atg proteins play an essential role in both types of autophagy. However, the *atg17*Δ/Δ strains exhibited weak autophagy under both conditions and seemed to show even weaker autophagy under nitrogen starvation (Figure 1), indicating that the importance of Atg17 in the two types of autophagy is different. The autophagy of these strains under an MMS treatment has been examined in our previous study [40], and the function of Atg1 and Atg11 on macroautophagy has also been identified [46,47]. However, in order to compare the differences between the two types of autophagy more intuitively, we repeated the experiment, and the results were consistent with our previous work [40,46,47]. These data indicated that the core Atg proteins (Atg1, Atg11, Atg13, and Atg18) have essential roles in DNA damage-induced autophagy and macroautophagy, while the function of Atg17 is slightly different between the two types of autophagy.

### 3.2. The Domains of Atg13 Play an Essential Role in DNA Damage-Induced Autophagy

**Figure 2:**
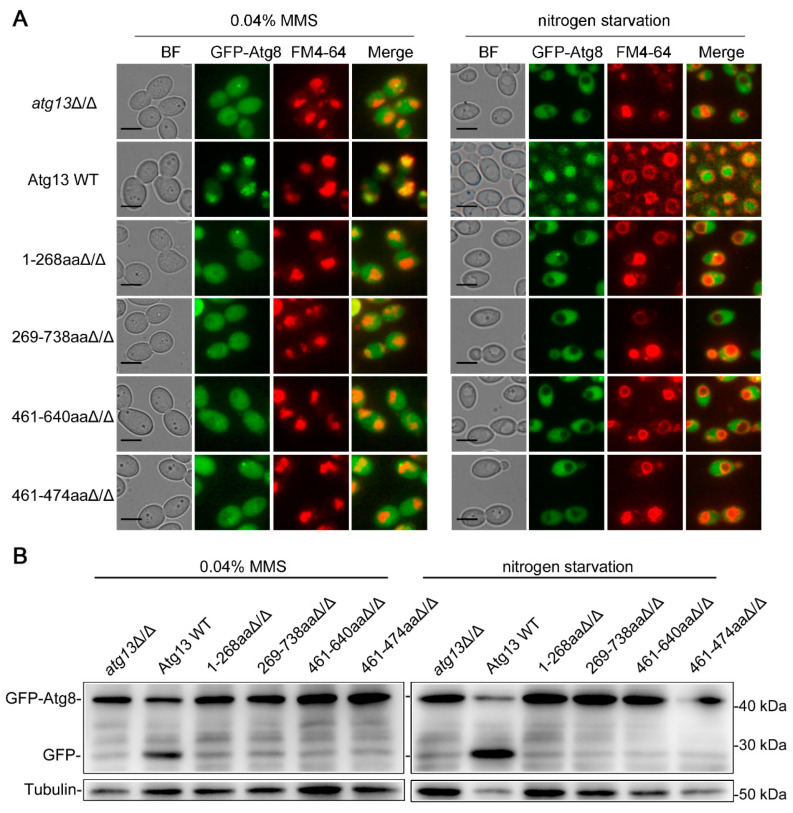
The domains of Atg13 play an essential role in DNA damage-induced autophagy and macroautophagy. (**A**) Localization of GFP-Atg8 under an MMS treatment and nitrogen starvation. GFP-Atg8 was expressed in related strains, and these strains were treated with MMS for 4 h or subjected to nitrogen starvation for 4 h. The cells were observed by fluorescence microscopy. BF, bright field. Scale bar = 5 μm. (**B**) Immunoblotting assay for GFP-Atg8 cleavage. Immunoblots were performed using the GFP antibody. The levels of Tubulin served as loading controls.

Atg13 is an essential protein in the core autophagic machinery, and its phosphorylation level directly affects the formation of the Atg1 complex, which is crucial for the initiation of autophagy [48]. In *S. cerevisiae*, Atg13 contains two kinds of structures: an *N*-terminal HORMA domain and a C-terminal disordered region [49]. Both of these domains are essential for macroautophagy in *S. cerevisiae*, but their effect on macroautophagy and DNA damage-induced autophagy in *C. albicans* remains unclear. To investigate the role of the domains of Atg13 in these two types of autophagy, the *ATG13* fragments (including the *ATG13* promoter, ORF, and terminator sequences) lacking the HORMA domain (1-268aa) or disordered region (269-738aa) were transformed into the *atg13*Δ/Δ strains, and the degree of autophagy was determined by the well-established GFP-Atg8 processing assay under an MMS treatment and nitrogen starvation. The results showed that the strains lacking the two domains exhibited a similar degree of autophagy as the *atg13*Δ/Δ strains, and there was almost no vacuole aggregation of Atg8 (Figure 2A and Figure S2A) and GFP accumulation (Figure 2B) in these two strains under an MMS treatment and nitrogen starvation, suggesting that the two domains of Atg13 play an essential role in both types of autophagy in *C. albicans*. It has been proven that Atg13 461-474aa, a fragment in the C-terminal disordered region of Atg13, was not required for macroautophagy but was required for glucose starvation-induced autophagy in *S. cerevisiae* [48]. We also examined the role of Atg13 461-474aa and the larger Atg13 461-640aa in the two types of autophagy in *C. albicans*. The results showed that the absence of Atg13 461-474aa or Atg13 461-640aa almost completely blocks autophagy under two conditions (Figure 2 and Figure S2A), suggesting that Atg13 461-474aa and Atg13 461-640aa are required for DNA damage-induced autophagy and macroautophagy in *C. albicans*. Together, these data indicated that the *N*-terminal HORMA domain and C-terminal disordered region of Atg13 are essential for DNA damage-induced autophagy and macroautophagy in *C. albicans*, and the Atg13 461-474aa are also required for these two types of autophagy in *C. albicans*.

### 3.3. The Destruction of Mec1-Rad53 Signaling Inhibits DNA Damage-Induced Autophagy

**Figure 3:**
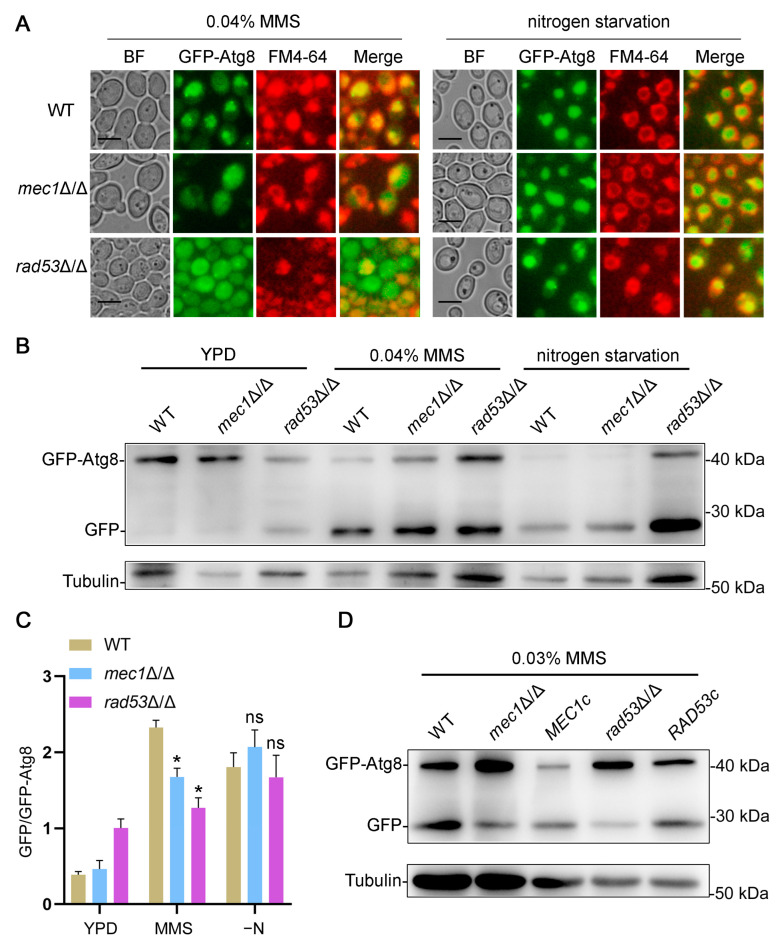
Mec1-Rad53 signaling positively regulates DNA damage-induced autophagy. (**A**) Localization of GFP-Atg8 under an MMS treatment and nitrogen starvation. GFP-Atg8 was expressed in the WT, *mec1*Δ/Δ, and *rad53*Δ/Δ strains, and these strains were treated with MMS for 4 h or subjected to nitrogen starvation for 4 h. The cells were observed with fluorescence microscopy. BF, bright field. Scale bar = 5 μm. (**B**) Immunoblotting assay for GFP-Atg8 cleavage. Immunoblots were performed using the GFP antibody. The levels of Tubulin served as loading controls. (**C**) The calculated ratio of GFP to GFP-Atg8. Data represent means ± SEM, *n* = 3. * *p* < 0.05, ns = not significant. (**D**) Immunoblotting assay for GFP-Atg8 cleavage. The *mec1*Δ/Δ and *rad53*Δ/Δ strains were transformed with either p*MEC1* or p*RAD53* and examined for autophagy under an MMS treatment.

When faced with DNA damage, cells will initiate the DNA damage response to protect themselves [3]. Mec1 and Rad53 are important components in the DNA damage response, and Rad53 is phosphorylated by Mec1 and acts downstream of Mec1 [10]. However, whether Mec1-Rad53 signaling regulates DNA damage-induced autophagy in *C. albicans* remains unclear. To explore whether Mec1-Rad53 signaling is involved in DNA damage-induced autophagy, GFP-Atg8 was transformed into strains lacking Mec1 or Rad53, and the well-established GFP-Atg8 processing assay was used to monitor autophagy under an MMS treatment and nitrogen starvation [44,45]. The degree of autophagy was represented by the degree of aggregation of GFP-Atg8 into the vacuole and the degree of transition from GFP-Atg8 to GFP. The results showed that under an MMS treatment, the deletion of Mec1 or Rad53 resulted in a decrease in the proportion of cells displaying vacuolar accumulation of GFP-Atg8 (Figure 3A and Figure S1B). Compared with the WT strains, the conversion of GFP-Atg8 into GFP in the *mec1*Δ/Δ and *rad53*Δ/Δ strains was significantly reduced under an MMS treatment (Figure 3B,C). We re-transferred *MEC1* and *RAD53* into the *mec1*Δ/Δ and *rad53*Δ/Δ strains, respectively, and found that the autophagy defects under an MMS treatment was successfully compensated (Figure 3D). These results suggested that Mec1 and Rad53 have a positive role in the regulation of DNA damage-induced autophagy. We also examined the autophagy of the *mec1*Δ/Δ and *rad53*Δ/Δ strains under nitrogen starvation, and we found that the absence of Mec1 or Rad53 did not affect autophagy under nitrogen starvation (Figure 3 and Figure S1B). These data indicated that Mec1-Rad53 signaling has no regulatory effect on macroautophagy and only regulates DNA damage-induced autophagy specifically.

### 3.4. The Destruction of Mec1-Rad53 Signaling Inhibits the Recruitment of Atg1 and Atg13 to PAS

**Figure 4:**
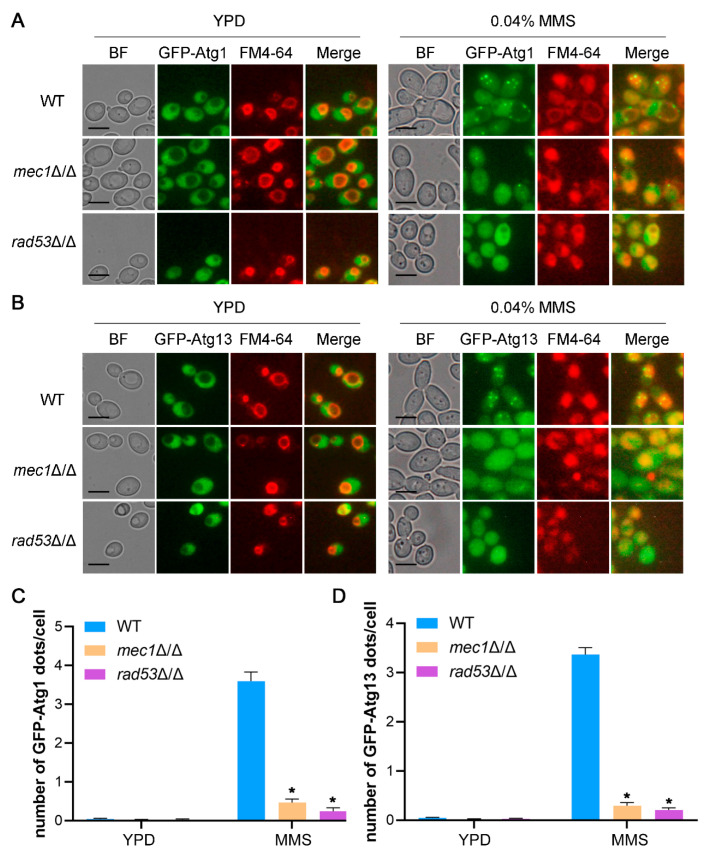
Mec1-Rad53 signaling affects the formation of Atg1 and Atg13 puncta under an MMS treatment. (**A**) Localization of GFP-Atg1 under an MMS treatment. GFP-Atg1 was expressed in the WT, *mec1*Δ/Δ, and *rad53*Δ/Δ strains, and these strains were treated with MMS for 4 h or not. The cells were observed by fluorescence microscopy. BF, bright field. Scale bar = 5 μm. (**B**) Localization of GFP-Atg13 under an MMS treatment. (**C**,**D**) Statistics on the number of GFP-Atg1 and GFP-Atg13 puncta under an MMS treatment. The number of GFP-Atg1 and GFP-Atg13 puncta in each cell was counted. More than 300 cells were counted. Data represent means ± SEM, *n* = 3. * *p* < 0.05.

**Figure 5:**
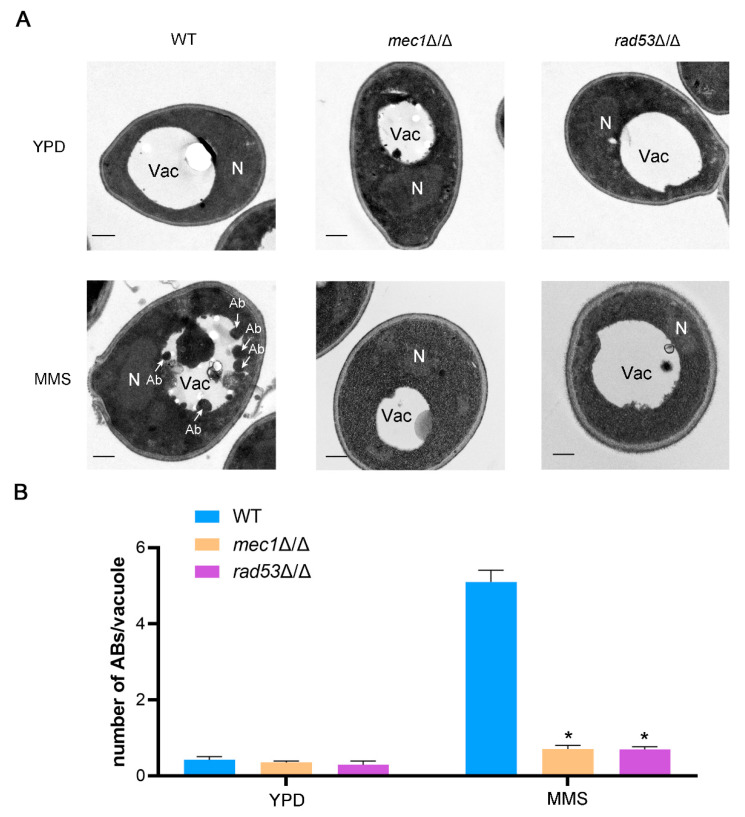
Mec1-Rad53 signaling affects the number of autophagic bodies. (**A**) Representative electron microscopic pictures of the WT, *mec1*Δ/Δ, and *rad53*Δ/Δ strains under YPD conditions and an MMS treatment. The arrow points to autophagic bodies. An amount of 1 mM PMSF was used to prevent autophagic bodies degradation. Scale bar = 500 nm. Ab: Autophagic body; As: Autophagosome; N: Nucleus. Vac: Vacuole. (**B**) Statistics on the number of autophagic bodies per cell. More than 100 cells were counted. Data represent means ± SEM, *n* = 3. * *p* < 0.05.

The autophagy initiation complex, Atg1 complex, consists of Atg1, Atg13, Atg17, Atg29, and Atg31, and when autophagy is activated, these proteins are gradually recruited into PAS, providing the initial structure for the formation of the phagophore [42,50]. Phosphorylation of Atg13 regulates the binding of these proteins, thereby affecting the serine/threonine kinase activity of Atg1, which is essential for the function of the Atg1 complex [42,50]. The correct localization of Atg1 and Atg13 plays a critical role in autophagy initiation and autophagosome formation [42]. To explore whether Mec1-Rad53 signaling regulates DNA damage-induced autophagy by affecting the recruitment of Atg1 and Atg13 to PAS, we labeled Atg1 and Atg13 with GFP at the amino terminal, and the localization of Atg1 and Atg13 was observed with fluorescence microscopy. The results showed that, under YPD conditions, GFP-Atg1 and GFP-Atg13 rarely formed puncta in the WT and mutant strains (Figure 4), suggesting that Atg1 and Atg13 were not recruited to PAS under YPD conditions. Under an MMS treatment, GFP-Atg1 and GFP-Atg13 formed puncta in the WT strains (Figure 4), suggesting that Atg1 and Atg13 in the WT strains were recruited to PAS. However, compared to the WT strains, the *mec1*Δ/Δ and *rad53*Δ/Δ strains exhibited fewer Atg1-GFP and Atg13-GFP puncta under an MMS treatment, suggesting that the absence of Mec1 or Rad53 affects the recruitment of Atg1 and Atg13 to PAS (Figure 4). The recruitment of Atg proteins to PAS is critical for the normal formation of autophagosomes, which directly affect the process of autophagy [42]. We also measured the number of autophagic bodies in the vacuoles using transmission electron microscopy (TEM), which are produced by the fusion of autophagosomes and vacuoles. The results showed that, under YPD conditions, these strains had almost no autophagic bodies in the vacuoles (Figure 5). Under an MMS treatment, the number of autophagic bodies in the vacuoles of the *mec1*Δ/Δ and *rad53*Δ/Δ strains was significantly less than that of the WT strains (Figure 5), suggesting that the absence of Mec1 and Rad53 may affect the formation of autophagosomes. Together, these data indicated that Mec1-Rad53 signaling may influence the formation of autophagosomes by affecting the recruitment of Atg1 and Atg13 to PAS, thus regulating autophagy.

### 3.5. DNA Damage Failed to Induce Mitophagy and ER Autophagy

**Figure 6:**
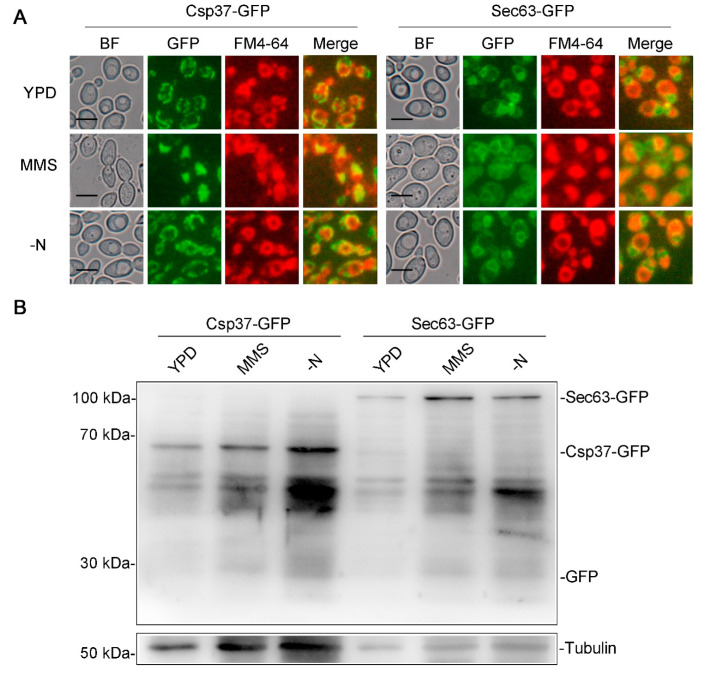
DNA damage does not induce mitophagy and ER autophagy. (**A**) Localization of Csp37-GFP and Sec63-GFP under an MMS treatment and nitrogen starvation. The wild-type strains containing Csp37-GFP or Sec63-GFP were treated with MMS and stained by FM4-64, and the cells were observed by fluorescence microscopy. BF, bright field. Scale bar = 5 μm. (**B**) Immunoblotting assay for Csp37-GFP and Sec63-GFP cleavage. Immunoblots were performed using the GFP antibody. The levels of Tubulin served as loading controls.

We have proven that DNA damage caused by MMS can induce autophagy in *C. albicans* [40], and DNA also exists in the mitochondria [51]. In *S. cerevisiae*, Om45 is a mitochondrial matrix protein, and its degradation can be used to represent the degree of mitophagy [52]. To explore whether DNA damage caused by MMS can induce mitophagy in *C. albicans*, we labeled Csp37 (a homolog of Om45 in *C. albicans*) with GFP at the carboxyl terminal in the WT strains, and the GFP accumulation can represent the degree of mitophagy. The results showed that under nitrogen starvation, the localization of Csp37 is similar to that under YPD conditions (Figure 6A), and there is no accumulation of GFP under either condition (Figure 6B), suggesting that mitophagy is not activated under either condition. Under an MMS treatment, the localization of Csp37 was affected compared with that under YPD conditions (Figure 6A), but there is no accumulation of GFP under this condition (Figure 6B), suggesting that DNA damage affected mitochondrial morphology but did not activate mitophagy. We also explored the effect of DNA damage on endoplasmic reticulum (ER) autophagy, another selective autophagy responsible for transporting damaged endoplasmic reticulum to the vacuole for degradation [53]. Sec63 is an ER marker protein, and its degradation level can represent the degree of ER autophagy [53]. The results showed that the morphology of ER did not change under these three conditions (Figure 6A), and there was no accumulation of GFP (Figure 6B), suggesting that DNA damage also did not activate ER autophagy.

### 3.6. Identification of Regulators in DNA Damage-Induced Autophagy

**Figure 7:**
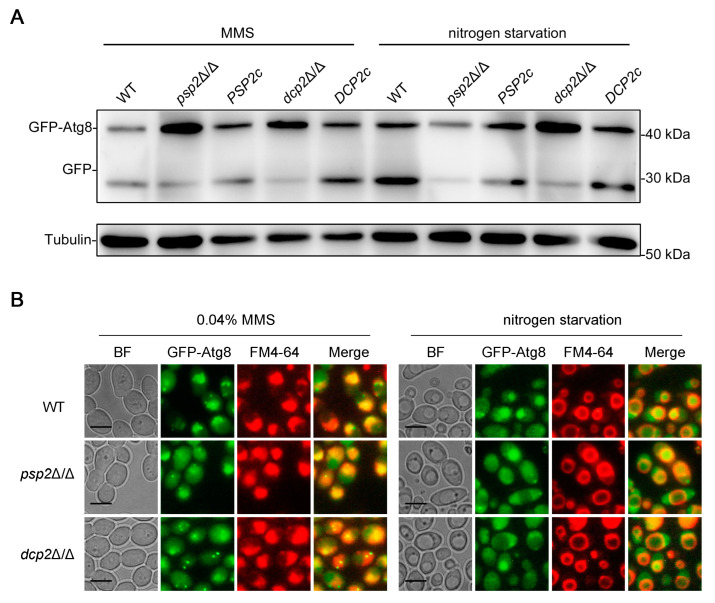
Deletion of Psp2 or Dcp2 inhibits DNA damage-induced autophagy and macroautophagy. (**A**) Immunoblotting assay for GFP-Atg8 cleavage. GFP-Atg8 was expressed in the WT, *psp2*Δ/Δ, *PSP2c*, *dcp2*Δ/Δ, and *DCP2c* strains, and these strains were treated with MMS for 4 h or subjected to nitrogen starvation for 2 h. Immunoblots were performed using the GFP antibody. The levels of Tubulin served as loading controls. (**B**) Localization of GFP-Atg8 under an MMS treatment and nitrogen starvation. The cells were observed with fluorescence microscopy. BF, bright field. Scale bar = 5 μm.

Autophagy is a rigorously coordinated and highly complex process, and its dysregulation is implicated in many human diseases, such as lysosomal storage diseases, cancer, and neurodegeneration [54]. Therefore, the autophagy process must be closely regulated to maintain the appropriate level. Psp2 is an RNA-binding protein, which regulates macroautophagy at the translational level in *S. cerevisiae* [55]. The decapping enzyme Dcp2 is responsible for removing the 5′-methylguanosine cap of the mRNA and promoting the degradation of mRNA, which regulates macroautophagy at the post-transcriptional level in *S. cerevisiae* [56]. However, the role of these regulators in DNA damage-induced autophagy and macroautophagy in *C. albicans* remains unclear. We constructed the strains lacking these regulators and measured their autophagy under an MMS treatment and nitrogen starvation using a well-established GFP-Atg8 processing assay. The results showed that these strains had no significant GFP accumulation under YPD conditions, and only the *dcp2*Δ/Δ strains had very little GFP accumulation (Figure 7A), suggesting that autophagy did not occur significantly in these strains under YPD conditions. Under an MMS treatment and nitrogen starvation, the *psp2*Δ/Δ and *dcp2*Δ/Δ strains exhibited less GFP accumulation and vacuole aggregation of Atg8 compared to the WT strains (Figure 7). We re-introduced the *PSP2* and *DCP2* into the *psp2*Δ/Δ and *dcp2*Δ/Δ strains, respectively, and found that the autophagy defects were successfully rescued (Figure 7A). These results suggested that the absence of Psp2 or Dcp2 inhibits DNA damage-induced autophagy and macroautophagy.

### 3.7. Psp2 and Dcp2 Regulates the Expression of Atg Proteins and DDR-Related Proteins

**Figure 8:**
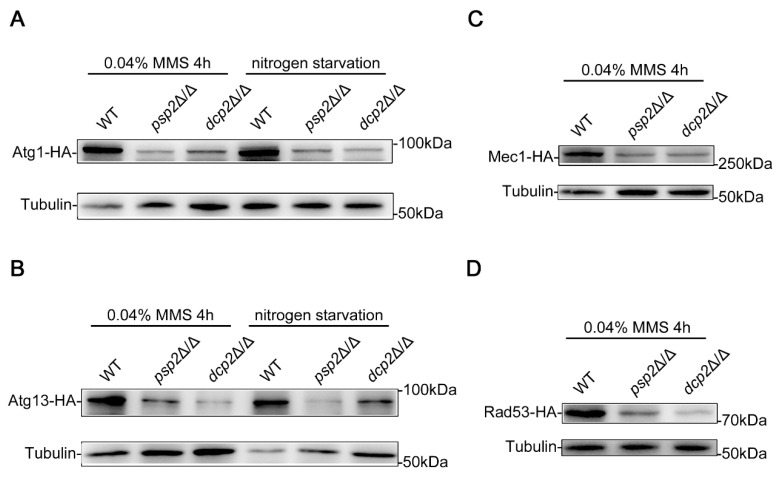
Psp2 and Dcp2 regulates the protein levels of Atg1, Atg13, Mec1, and Rad53. (**A**) Immunoblotting assay for the protein level of Atg1. (**B**) Immunoblotting assay for the protein level of Atg13. (**C**,**D**) Immunoblotting assay for the protein level of Mec1 and Rad53. The WT, *psp2*Δ/Δ, and *dcp2*Δ/Δ strains containing Atg1-HA, Atg13-HA, Mec1-HA, or Rad53-HA were treated with MMS for 4 h or subjected to nitrogen starvation for 2 h, and immunoblots were performed using the HA antibody. The levels of Tubulin served as loading controls.

Both Psp2 and Dcp2 are RNA-binding proteins, in which Psp2 regulates mRNA translation and Dcp2 promotes mRNA degradation [55,56], both of which have a direct impact on protein expression. Atg1 and Atg13 are core proteins that regulate autophagy [16], and the absence of Atg1 or Atg13 completely blocks DNA damage-induced autophagy and macroautophagy in *C. albicans* (Figure 1). To explore whether Psp2 and Dcp2 regulate DNA damage-induced autophagy and macroautophagy through affecting the expression of Atg proteins, we labeled Atg1 and Atg13 in situ at the carboxyl terminus using HA tags and measured the protein levels of Atg1 and Atg13 with Western blotting under an MMS treatment and nitrogen starvation. The results showed that the protein levels of Atg1 and Atg13 were significantly reduced in the *psp2*Δ/Δ and *dcp2*Δ/Δ strains compared to the WT strains under an MMS treatment (Figure 8A), and there is a similar situation under nitrogen starvation (Figure 8B), suggesting that Psp2 and Dcp2 indeed regulate DNA damage-induced autophagy and macroautophagy by affecting the protein levels of Atg1 and Atg13. The deletion of Mec1 or Rad53 significantly inhibits DNA damage-induced autophagy (Figure 3), and we also explored whether Psp2 and Dcp2 regulate DNA damage-induced autophagy by affecting the Mec1-Rad53 signaling. We measured the protein levels of Mec1 and Rad53 in the same way and found that the absence of Psp2 or Dcp2 significantly reduces the protein levels of Mec1 and Rad53 (Figure 8C,D). Together, these data indicated that Psp2 and Dcp2 regulate autophagy by affecting the expression of Atg proteins and DDR-related proteins.

### 3.8. The Destruction of Mec1-Rad53 Signaling Attenuates the Virulence of C. albicans

**Figure 9:**
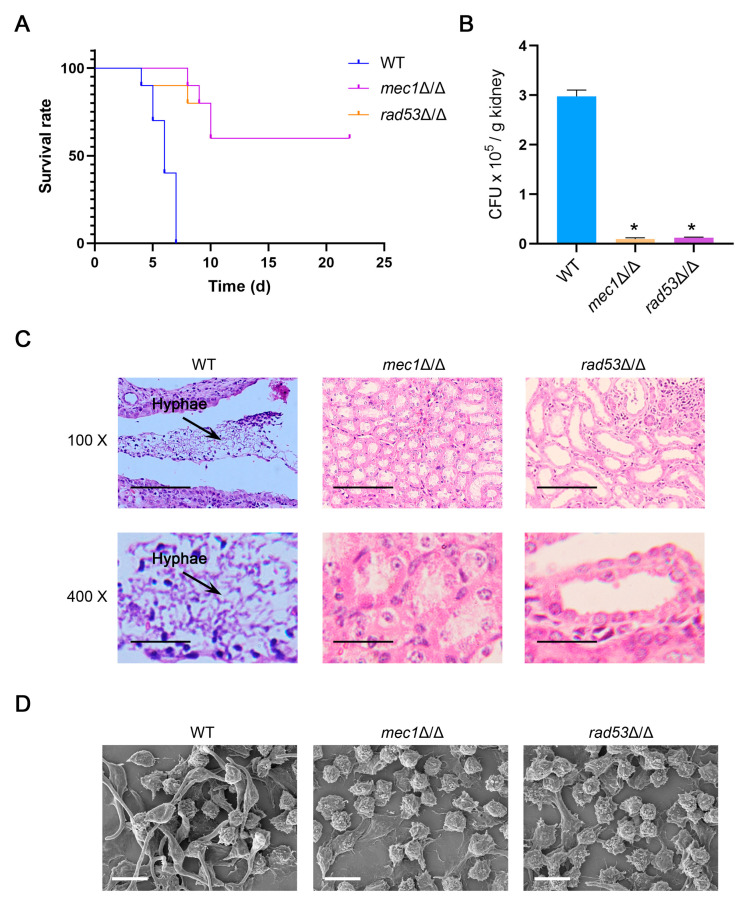
Mec1-Rad53 signaling is required for the virulence of *C. albicans*. (**A**) Survival curves of mice infected by the WT, *mec1*Δ/Δ, and *rad53*Δ/Δ strains. Each group of mice was monitored for more than 20 days, and their survival rates were recorded. (**B**) Statistics of fungal burdens of the kidneys in the injected mice. Kidneys were removed from sacrificed mice and plated on YPD plates. The number of colonies was counted. Data represent means ± SEM, *n* = 3. * *p* < 0.05. (**C**) Histopathological observation of kidneys from infected mice. After being removed from sacrificed mice, the kidneys were fixed and stained, and microscopy was used to observe the stained sections. The arrows point to the hyphae invading the kidneys. Scale bar (top), 100 μm. Scale bar (low), 25 μm. (**D**) Observation of the interaction between *C. albicans* and macrophages. *C. albicans* and macrophages were co-cultured at 37 °C for 1 h 40 min; after being fixed and gradient dehydrated, the samples were observed with a scanning electron microscope (SEM). Scale bar, 10 μm.

It has been proven that the DNA damage response plays a critical role in the virulence of *C. albicans* [35]. For example, the deletion of Rtt109 increases sensitivity to DNA damage stress agents in *C. albicans* and significantly reduces the virulence in the murine model [36]. The *rad52*Δ/Δ strains exhibit reduced virulence in a murine model [37]. However, whether the Mec1-Rad53 signaling regulates the virulence of *C. albicans* remains unclear. Since *URA3* has an essential role in the virulence of *C. albicans* [57], we retransformed the *URA3* gene into the *mec1*Δ/Δ, *rad53*Δ/Δ, and WT strains and tested these strains for a systemic infection in the mice. The results showed that the *mec1*Δ/Δ and *rad53*Δ/Δ strains exhibited significantly attenuated virulence compared to the WT strains (Figure 9A), suggesting that Mec1-Rad53 signaling regulates the virulence of *C. albicans*. The fungal load in the kidneys of mice infected with the *mec1*Δ/Δ and *rad53*Δ/Δ strains was significantly lower than that of mice infected with the WT strains (Figure 9B). A histological analysis further showed that the kidneys of mice infected by the WT strains were seriously hyphal infected, while the kidneys of mice injected with the *mec1*Δ/Δ and *rad53*Δ/Δ strains exhibited only slight infiltration and no hyphae (Figure 9C). When *C. albicans* infects the host, it will be recognized and attacked by the host’s immune system, and the phagocytosis of macrophages is a critical mechanism to kill *C. albicans* [58]. We also examined the efficacy of macrophages against these strains. *C. albicans* and macrophages were co-cultured at 37 °C, and the morphology of *C. albicans* was observed with a scanning electron microscope (SEM) after dehydration and drying. Our results showed that under the action of macrophages, the WT strains still have a lot of hyphae, while the *mec1*Δ/Δ and *rad53*Δ/Δ strains have almost no hyphae (Figure 9D), suggesting that the killing effect of macrophages on *mec1*Δ/Δ and *rad53*Δ/Δ was stronger. Together, these data indicated that Mec1-Rad53 signaling plays a positive role in the virulence of *C. albicans*.

## 4. Discussion

DNA damage can induce autophagy in yeast and mammalian cells [21,22], and in our previous work, we identified the DNA damage-induced autophagy in *C. albicans* [40]. Atg proteins are directly involved in the regulation of autophagy [16,17,18,19,20], but whether their roles are different in DNA damage-induced autophagy and macroautophagy remains unclear. We found that Atg1, Atg11, Atg13, and Atg18 play an essential role in the two types of autophagy in *C. albicans* (Figure 1), and Atg17 also plays a role in these processes, but this role is not essential (Figure 1). Compared with DNA damage-induced autophagy, Atg17 has a more important function in macroautophagy (Figure 1). In *S. cerevisiae*, Atg17 formed a subcomplex with Atg29 and Atg31, then recruited Atg1 and Atg13 to form the Atg1 complex to induce macroautophagy initiation [43]. Atg17 is essential for glucose starvation-induced autophagy but not for macroautophagy in *S. cerevisiae* [43]. We speculated that core Atg proteins, such as Atg1, Atg11, and Atg13, are conserved in different types of autophagy and even in different species, but the function of non-core Atg proteins, such as Atg17, may change to some extent, and their function may be shared by other Atg proteins. We also measured the effect of the *N*-terminal HORMA domain and the C-terminal disordered region of Atg13 on DNA damage-induced autophagy and macroautophagy, and we found that these two domains are essential for these two types of autophagy (Figure 2). We also found that the fragment 461-474aa in the C-terminal disordered region of Atg13 has an essential role in the two types of autophagy (Figure 2). In *S. cerevisiae*, the fragment 461-474aa is only required for glucose starvation-induced autophagy and not for macroautophagy [48]. We speculated that the fragment 461-474aa might have become more functional over the course of evolution.

We explored the effect of the Mec1-Rad53 signaling pathway, a key pathway in the DNA damage response, on DNA damage-induced autophagy and macroautophagy, and we found that Mec1-Rad53 signaling specifically regulates DNA damage-induced autophagy and has no effect on macroautophagy in *C. albicans* (Figure 3). In *S. cerevisiae*, during glucose starvation-induced autophagy, Mec1 regulates the recruitment of Atg1-Atg13 to PAS by directly binding with Atg1-Atg13 [48]. In the present study, we found that the destruction of Mec1-Rad53 signaling significantly inhibits the formation of Atg1 and Atg13 puncta under an MMS treatment (Figure 4) and leads to a decrease in the number of autophagic bodies in the vacuole (Figure 5). We speculated that Mec1 might also affect the recruitment of Atg1-Atg13 to PAS by directly binding with Atg1 and Atg13, which further influences the assembly of the Atg1 complex and the formation of autophagosomes, and it ultimately regulates DNA damage-induced autophagy. We also found that DNA damage does not induce mitophagy and ER autophagy (Figure 6), indicating that DNA damage has no direct relationship with these two types of selective autophagy.

We identified regulators in DNA damage-induced autophagy that function at the translational and post-transcriptional levels, respectively (Figure 7). We focused on the regulatory functions of translational regulator Psp2 and post-transcriptional regulator Dcp2 and found that Psp2 and Dcp2 positively regulate DNA damage-induced autophagy and macroautophagy by affecting the protein levels of Atg proteins (Atg1 and Atg13) and DDR-related proteins (Mec1 and Rad53) (Figure 7 and Figure 8). In *S. cerevisiae*, Psp2 interacts with the components eIF4E and eIF4G2 in the translation initiation machinery and binds directly to the mRNA to regulate the translation process of the mRNA [55]. Therefore, we speculated that Psp2 might directly bind to *ATG1* and *ATG13* mRNA to regulate the protein levels of Atg1 and Atg13 under an MMS treatment and nitrogen starvation. Under an MMS treatment, Psp2 might also directly bind to *MEC1* and *RAD53* mRNA to regulate the protein levels of Mec1 and Rad53. In *S. cerevisiae*, the decapping enzyme Dcp2 promotes the degradation of *ATG* mRNAs by removing the 5′-methylguanosine caps of the *ATG* mRNAs to maintain autophagy activity at a basal level under nutrient-rich conditions [56], and the absence of Dcp2 leads to enhanced autophagy under nitrogen starvation [59], which seems inconsistent with our results. Dhh1, a DExD/H-box RNA helicase, conveys *ATG* mRNAs to the decapping machinery for degradation, which also maintains autophagy activity at a basal level by limiting the amount of mRNA [59]. However, in addition to its inhibition of autophagy in nutrient-rich conditions, Dhh1 also promotes the translation of *ATG1* and *ATG13* mRNAs to induce autophagy under nitrogen starvation [60]. Therefore, we speculated that in addition to the negative regulation of autophagy at the post-transcriptional level, Dcp2 may also play a positive role in autophagy by promoting the translation of *ATG1* and *ATG13* mRNAs under an MMS treatment and nitrogen starvation. We demonstrated that the Mec1-Rad53 signaling positively regulates the pathogenicity of *C. albicans* through a systemic infection in mice and an interaction between *C. albicans* and macrophages. Mec1 is responsible for the phosphorylation and activation of Rad53, and we found that the deletion of Mec1 significantly attenuates the virulence of *C. albicans* (Figure 9). Pph3 is involved in deactivating Rad53 in *C. albicans*, and the absence of Pph3 enhances the pathogenicity of *C. albicans* [39]. These results indicated that the activation of Rad53 plays a critical role in the virulence of *C. albicans*, and our data enriched the mechanisms by which the DNA damage response regulates the pathogenicity of *C. albicans*.

In summary, we compared the function of Atg proteins in DNA damage-induced autophagy and macroautophagy and found that the core Atg proteins are conserved in these two types of autophagy, but Atg17 is slightly different. We also found that Mec1-Rad53 signaling specifically regulates DNA damage-induced autophagy and affects the formation of autophagosomes by influencing the recruitment of Atg1 and Atg13 to PAS. Our results showed that DNA damage does not induce mitophagy and ER autophagy. We identified the functions of regulators Psp2 and Dcp2 in DNA damage-induced autophagy and macroautophagy and found that Psp2 and Dcp2 positively regulates these two types of autophagy by affecting the protein levels of Atg1 and Atg13. The protein levels of Mec1 and Rad53 are also regulated by Psp2 and Dcp2 under an MMS treatment. We also found that Mec1-Rad53 signaling plays a positive role in the pathogenicity of *C. albicans*.

## Data Availability

The data that support the findings of this study are available from the corresponding author (nklimingchun@163.com) upon reasonable request.

## Supplementary Materials

The following supporting information can be downloaded at: https://www.mdpi.com/article/10.3390/jof9121181/s1, File S1: Molecular characterization of strains; Figure S1: (A) Localization of GFP-Atg8 under YPD conditions. GFP-Atg8 was expressed in WT, *mec1*Δ/Δ, *rad53*Δ/Δ strains, and these strains were stained by FM4-64 and observed by fluorescence microscopy. BF, bright field. Scale bar = 5 μm. (B) Percentage of cells with vacuolar accumulated GFP-Atg8. More than 300 cells were counted. Data represent means ± SEM, n = 3. * *p* < 0.05, ns = not significant; Figure S2: (A) Percentage of Atg13 domains deletion related strains with vacuolar accumulated GFP-Atg8. (B) Immunoblotting assay for GFP-Atg8 cleavage under YPD conditions. (C) Percentage of autophagy regulators deletion related strains with vacuolar accumulated GFP-Atg8. More than 300 cells were counted. Data represent means ± SEM, n = 3. * *p* < 0.05, ns = not significant; Table S1: Strains and plasmids used in this study; Table S2: Primers used in this study.

## Abbreviations

DDR: DNA damage response; *ATG*, autophagy-related; GFP, green fluorescent protein; PAS, phagophore assembly sites; MMS, methyl methanesulfonate; WT, wild-type.
