# Supplementary material for: Mec1-Rad53 Signaling Regulates DNA Damage-Induced Autophagy and Pathogenicity in Candida albicans
Source: J Fungi (Basel). 2023 Dec 9;9(12):1181. doi: 10.3390/jof9121181 (PMC10744610; doi:10.3390/jof9121181)
Supplement: Supplementary file 1 [file jof-09-01181-s001.zip › FigureS1.pdf]

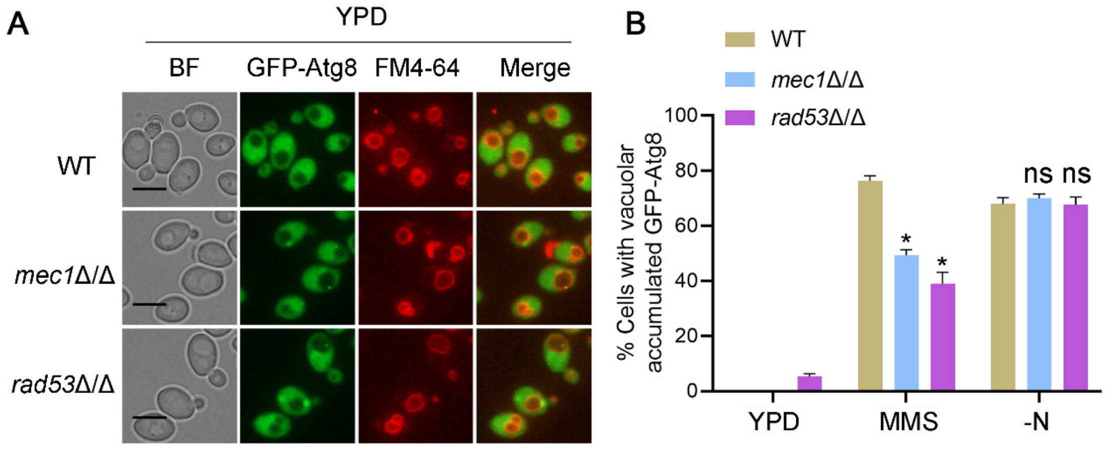

Fig. S1. (A) Localization of GFP-Atg8 under YPD conditions. GFP-Atg8 was expressed in WT, *mec1*Δ/Δ, *rad53*Δ/Δ strains, and these strains were stained by FM4-64 and observed by fluorescence microscopy. BF, bright field. Scale bar = 5 μm. (B) Percentage of cells with vacuolar accumulated GFP-Atg8. More than 300 cells were counted. Data represent means ± SEM, n = 3. $*p < 0.05$, ns = not significant.
