# Supplementary material for: Mec1-Rad53 Signaling Regulates DNA Damage-Induced Autophagy and Pathogenicity in Candida albicans
Source: J Fungi (Basel). 2023 Dec 9;9(12):1181. doi: 10.3390/jof9121181 (PMC10744610; doi:10.3390/jof9121181)
Supplement: Supplementary file 1 [file jof-09-01181-s001.zip › FigureS2.pdf]

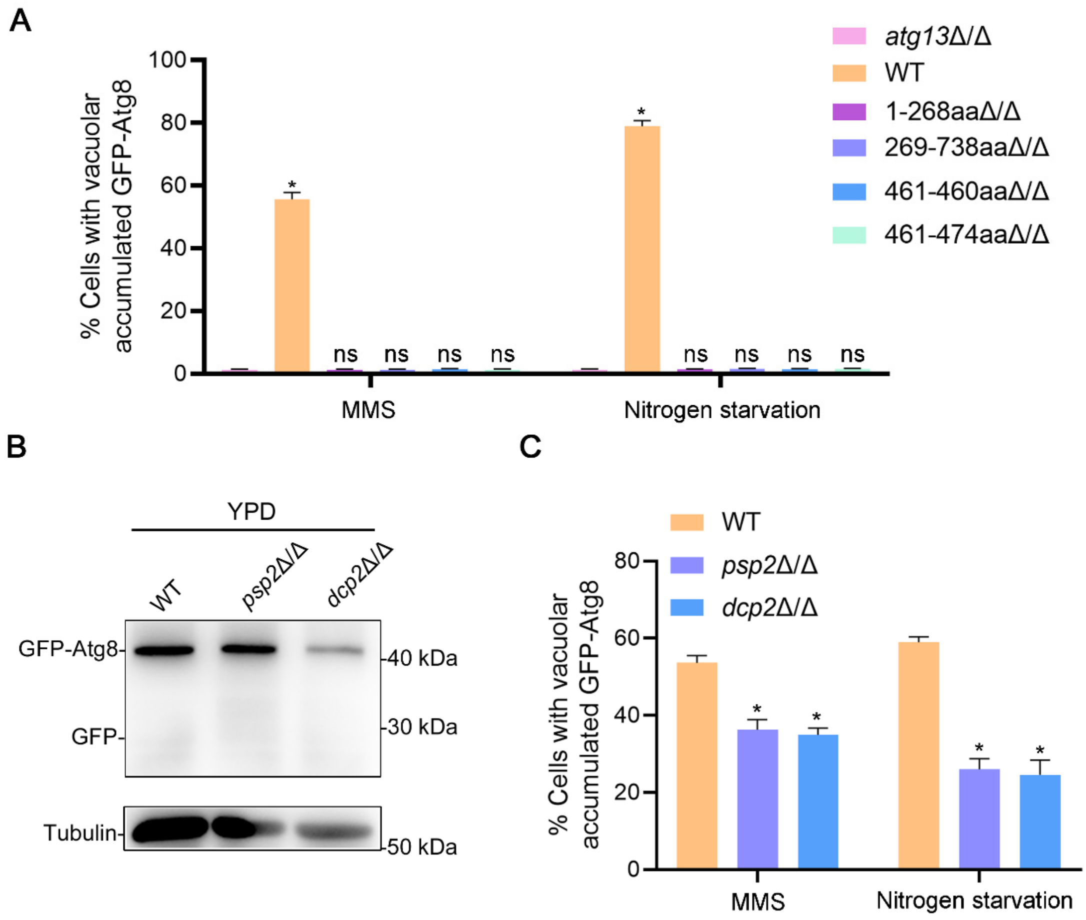


**Fig. S2.** (A) Percentage of Atg13 domains deletion related strains with vacuolar accumulated GFP-Atg8. (B) Immunoblotting assay for GFP-Atg8 cleavage under YPD conditions. (C) Percentage of autophagy regulators deletion related strains with vacuolar accumulated GFP-Atg8. More than 300 cells were counted. Data represent means ± SEM, n = 3. *$p < 0.05$, ns = not significant.
