# Supplementary material for: Mec1-Rad53 Signaling Regulates DNA Damage-Induced Autophagy and Pathogenicity in Candida albicans
Source: J Fungi (Basel). 2023 Dec 9;9(12):1181. doi: 10.3390/jof9121181 (PMC10744610; doi:10.3390/jof9121181)

Molecular characterization of strains

1. Molecular characterization of *mec1*Δ/Δ strains

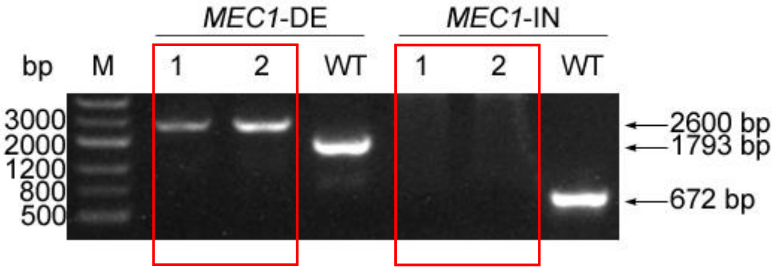

2. Molecular characterization of *MEC1c* strains (reintegrant control strains)

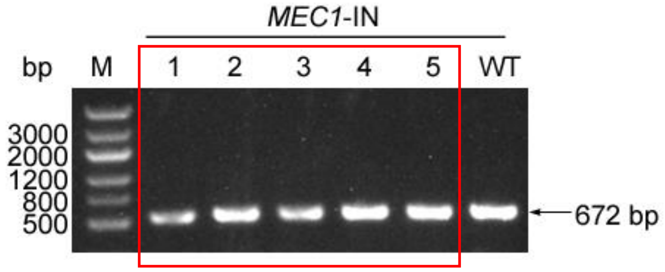

3. Molecular characterization of *rad53*Δ/Δ strains

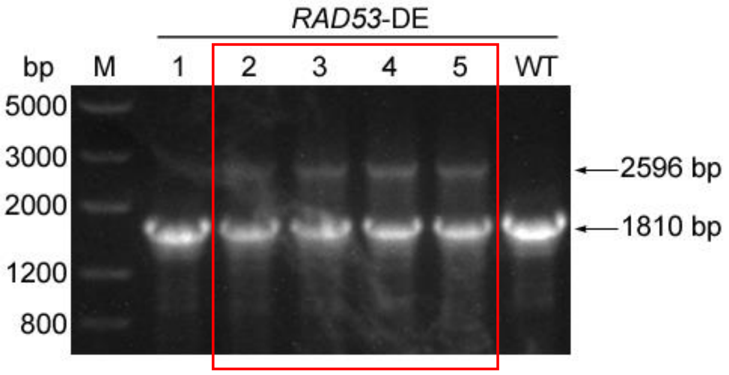

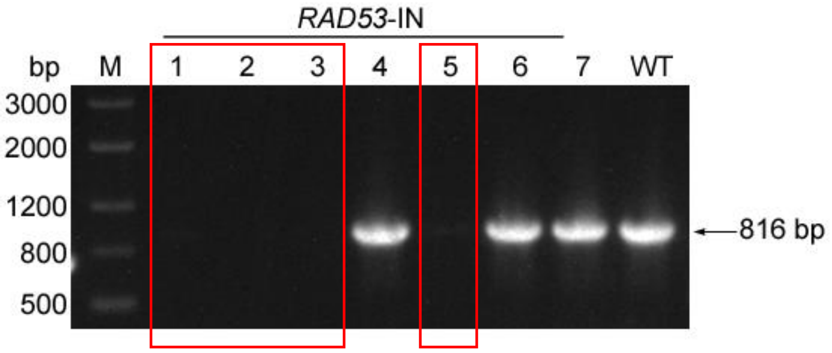

4. Molecular characterization of *RAD53c* strains (reintegrant control strains)

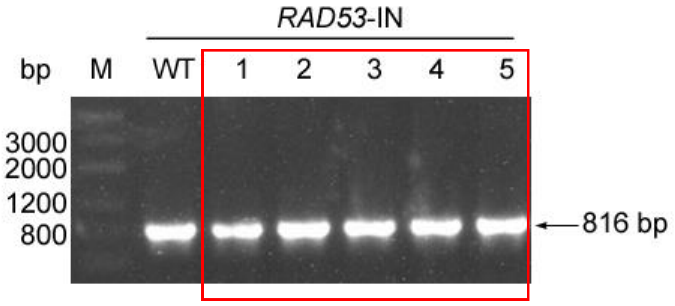

5. Double enzyme digestion of *ATG13* truncated plasmids

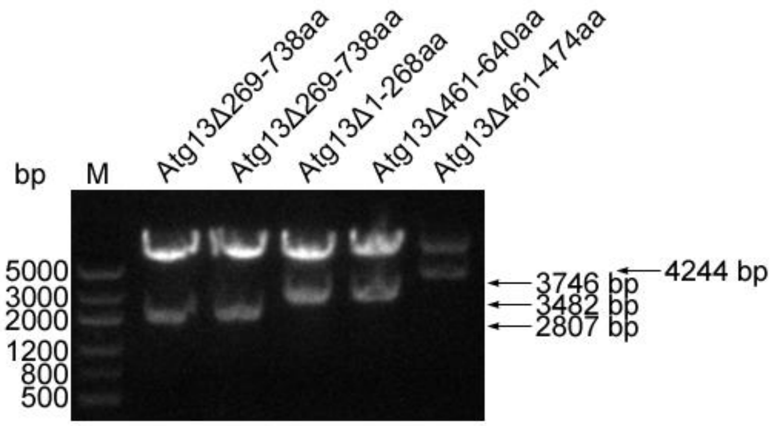

6. Molecular characterization of *ATG13* truncated strains

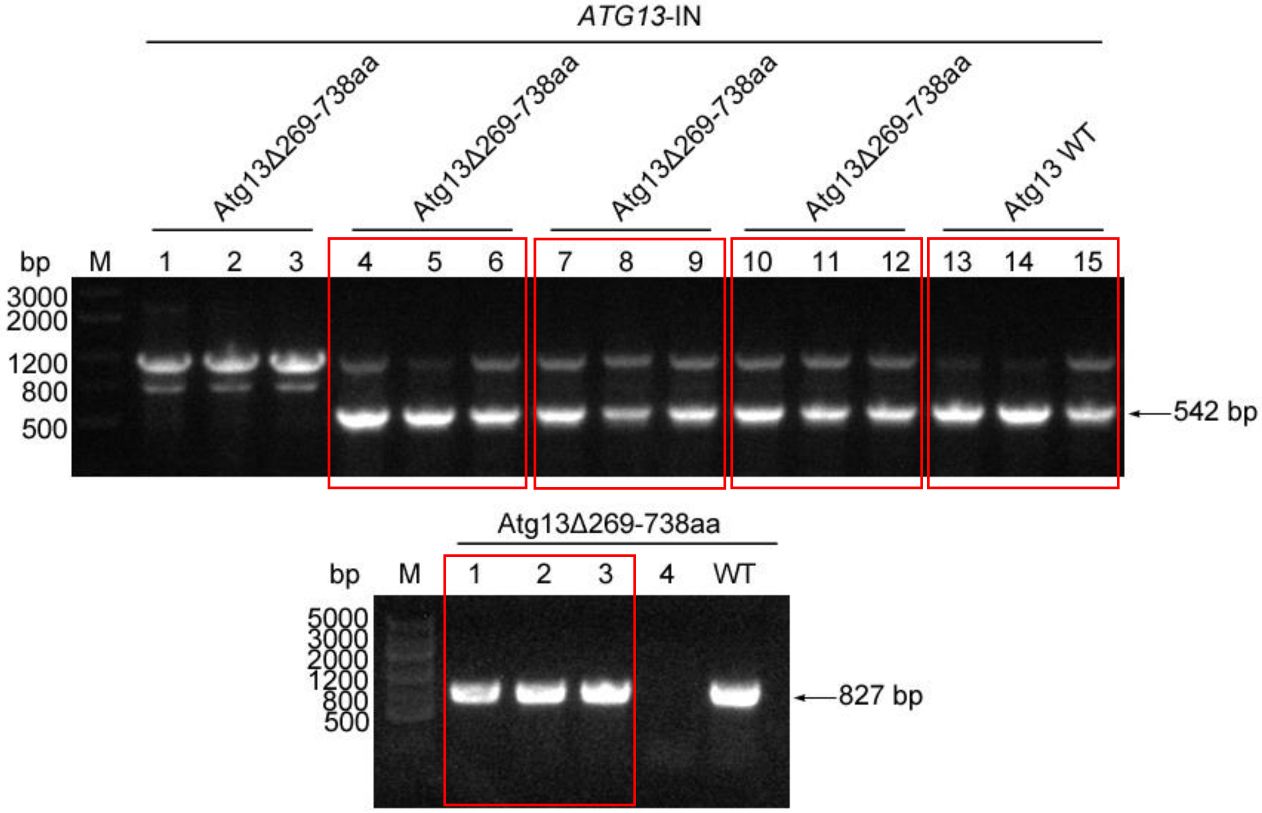

7. Molecular characterization of *psp2*Δ/Δ strains

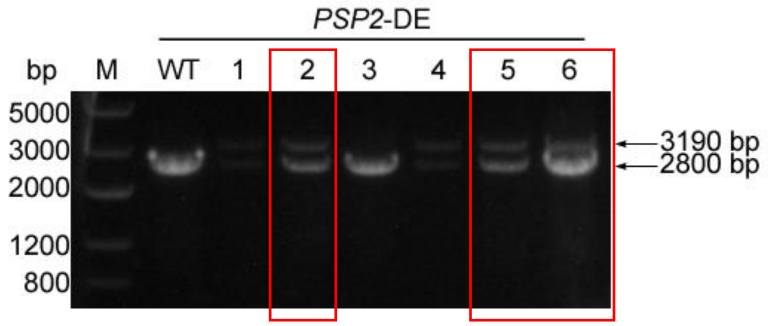

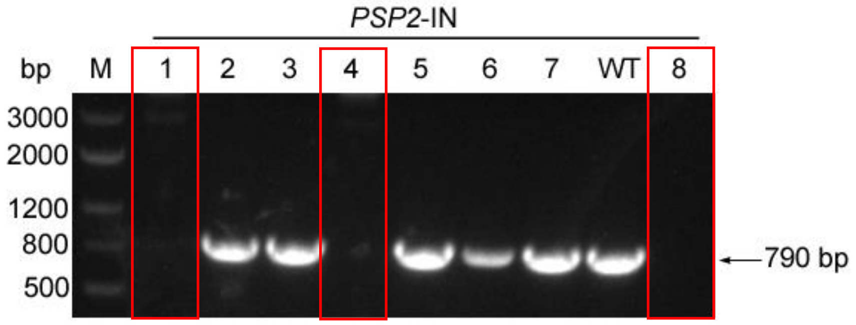

8. Molecular characterization of *PSP2c* strains (reintegrant control strains)

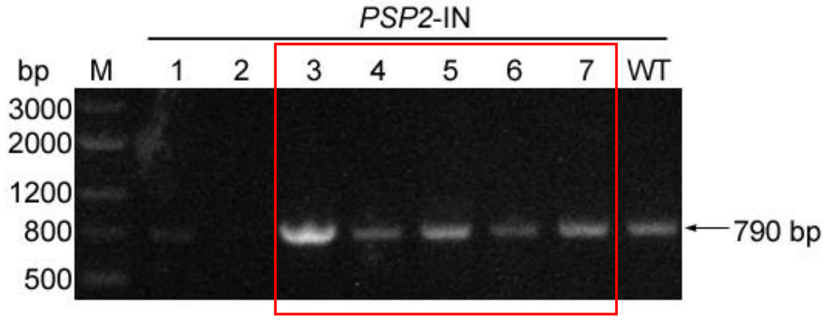

9. Molecular characterization of *dcp2*Δ/Δ strains

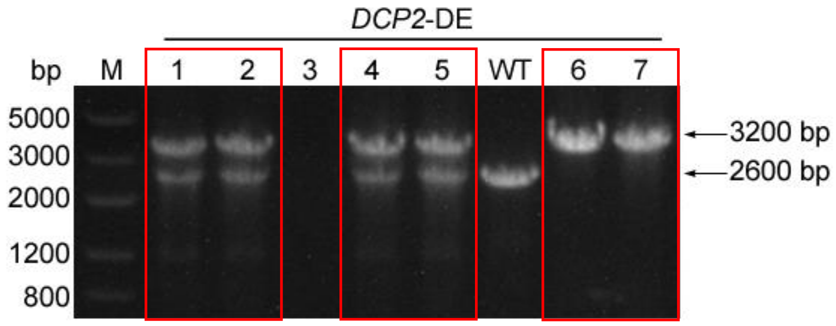

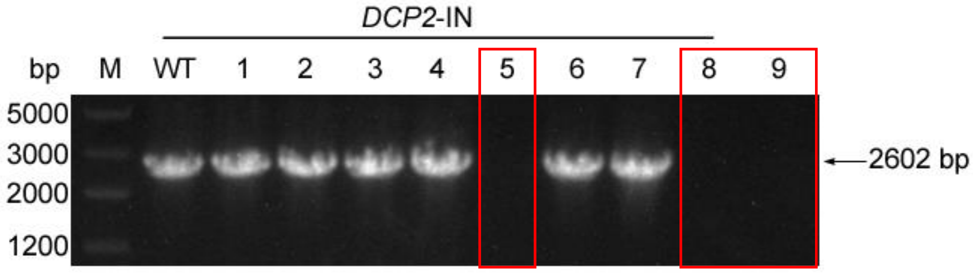

10. Molecular characterization of *DCP2c* strains (reintegrant control strains)

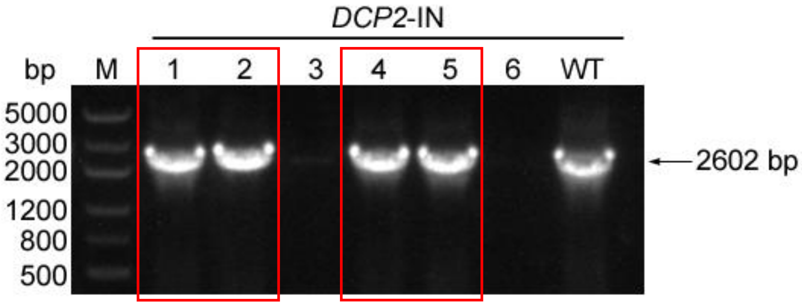

Supplement: Supplementary file 1 [file jof-09-01181-s001.zip › Molecular characterization of strains.pdf]
