# Supplementary material for: Mec1-Rad53 Signaling Regulates DNA Damage-Induced Autophagy and Pathogenicity in Candida albicans
Source: J Fungi (Basel). 2023 Dec 9;9(12):1181. doi: 10.3390/jof9121181 (PMC10744610; doi:10.3390/jof9121181)
Supplement: Supplementary file 1 [file jof-09-01181-s001.zip › TableS1.pdf]

Table S1 Strains and plasmids used in this study.

Strains or Plasmids	Genotype and description	Source
C. albicans **strains**		
WT (BWP17)	*ura3Δ::λimm434/ura3Δ::λimm434*<br>*his1::hisG/his1::hisG arg4::hisG/arg4::hisG*	Dana Davis
WT+GFP-*ATG8*	*ura3Δ::λimm434/ura3Δ::λimm434*<br>*his1::hisG/his1::hisG arg4::hisG/arg4::hisG*<br>*ACT1/ACT1::* P_{ACT1}*-GFP-ATG8*	Qilin Yu
*atg1*Δ/Δ+GFP-*ATG8*	*ura3Δ::λimm434/ura3Δ::λimm434*<br>*his1::hisG/his1::hisG arg4::hisG/arg4::hisG*<br>*atg1::ARG4/atg1:: URA3-dpl200*<br>*ACT1/ACT1::* P_{ACT1}*-GFP-ATG8*	Qilin Yu
*atg11*Δ/Δ+GFP-*ATG8*	*ura3Δ::λimm434/ura3Δ::λimm434*<br>*his1::hisG/his1::hisG arg4::hisG/arg4::hisG*<br>*atg11::ARG4/atg11:: URA3-dpl200*<br>*ACT1/ACT1::* P_{ACT1}*-GFP-ATG8*	Lifang Cui
*atg13*Δ/Δ+GFP-*ATG8*	*ura3Δ::λimm434/ura3Δ::λimm434*<br>*his1::hisG/his1::hisG arg4::hisG/arg4::hisG*<br>*atg13::ARG4/atg13:: URA3-dpl200*<br>*ACT1/ACT1::* P_{ACT1}*-GFP-ATG8*	Yixuan Dong
*atg17*Δ/Δ+GFP-*ATG8*	*ura3Δ::λimm434/ura3Δ::λimm434*<br>*his1::hisG/his1::hisG arg4::hisG/arg4::hisG*	Yixuan Dong

	atg17::ARG4/atg17:: URA3-dpl200<br>*ACT1/ACT1::* P_{ACT1}*-GFP-ATG8*	
*atg18*Δ/Δ+GFP-*ATG8*	*ura3Δ::λimm434/ura3Δ::λimm434*<br>*his1::hisG/his1::hisG arg4::hisG/arg4::hisG*<br>*atg18::ARG4/atg18:: URA3-dpl200*<br>*ACT1/ACT1::* P_{ACT1}*-GFP-ATG8*	Yixuan Dong
*atg13*Δ/Δ+*ATG13*+GFP-*ATG8*	*ura3Δ::λimm434/ura3Δ::λimm434*<br>*his1::hisG/his1::hisG arg4::hisG/arg4::hisG*<br>*atg13::ARG4/atg13:: URA3-dpl200*<br>*his1::hisG/his1::*P_{ATG13}*-ATG13*<br>*ACT1/ACT1::* P_{ACT1}*-GFP-ATG8*	This study
*atg13*Δ/Δ+*ATG13*$^{1-268aa\Delta/\Delta}$+GFP-*ATG8*	*ura3Δ::λimm434/ura3Δ::λimm434*<br>*his1::hisG/his1::hisG arg4::hisG/arg4::hisG*<br>*atg13::ARG4/atg13:: URA3-dpl200*<br>*his1::hisG/his1::*P_{ATG13}*-ATG13*$^{1-268aa\Delta/\Delta}$<br>*ACT1/ACT1::* P_{ACT1}*-GFP-ATG8*	This study
*atg13*Δ/Δ+*ATG13*$^{269-738\Delta/\Delta}$+GFP-*ATG8*	*ura3Δ::λimm434/ura3Δ::λimm434*<br>*his1::hisG/his1::hisG arg4::hisG/arg4::hisG*<br>*atg13::ARG4/atg13:: URA3-dpl200*<br>*his1::hisG/his1::*P_{ATG13}*-ATG13*$^{269-738aa\Delta/\Delta}$<br>*ACT1/ACT1::* P_{ACT1}*-GFP-ATG8*	This study
*atg13*Δ/Δ+*ATG13*$^{461-}$	*ura3Δ::λimm434/ura3Δ::λimm434*	This study

$^{640aa\Delta/\Delta}$+GFP-*ATG8*	*his1::hisG/his1::hisG arg4::hisG/arg4::hisG*<br>*atg13::ARG4/atg13:: URA3-dpl200*<br>*his1::hisG/his1::*P_{ATG13}*-ATG13*$^{461\text{-}640aa\Delta/\Delta}$<br>*ACT1/ACT1::* P_{ACT1}*-GFP-ATG8*	
atg13Δ/Δ+ATG13$^{461\text{-}474aa\Delta/\Delta}$+GFP-*ATG8*	*ura3Δ::λimm434/ura3Δ::λimm434*<br>*his1::hisG/his1::hisG arg4::hisG/arg4::hisG*<br>*atg13::ARG4/atg13:: URA3-dpl200*<br>*his1::hisG/his1::*P_{ATG13}*-ATG13*$^{461\text{-}474aa\Delta/\Delta}$<br>*ACT1/ACT1::* P_{ACT1}*-GFP-ATG8*	This study
mec1Δ/Δ-GFP-*ATG8*	*ura3Δ::λimm434/ura3Δ::λimm434*<br>*his1::hisG/his1::hisG arg4::hisG/arg4::hisG*<br>*mec1::ARG4/mec1:: URA3-dpl200*<br>*ACT1/ACT1::* P_{ACT1}*-GFP-ATG8*	This study
MEC1c-GFP-*ATG8*	*ura3Δ::λimm434/ura3Δ::λimm434*<br>*his1::hisG/his1::hisG arg4::hisG/arg4::hisG*<br>*mec1::ARG4/mec1:: URA3-dpl200*<br>*his1::hisG/his1::* P_{MEC1}*-MEC1*<br>*ACT1/ACT1::* P_{ACT1}*-GFP-ATG8*	This study
rad53Δ/Δ-GFP-*ATG8*	*ura3Δ::λimm434/ura3Δ::λimm434*<br>*his1::hisG/his1::hisG arg4::hisG/arg4::hisG*<br>*rad53::ARG4/rad53:: URA3-dpl200*<br>*ACT1/ACT1::* P_{ACT1}*-GFP-ATG8*	This study

RAD53c-GFP-*ATG8*	*ura3Δ::λimm434/ura3Δ::λimm434*<br>*his1::hisG/his1::hisG arg4::hisG/arg4::hisG*<br>*rad53::ARG4/rad53:: URA3-dpl200*<br>*his1::hisG/his1::* P_{RAD53}*-RAD53*<br>*ACT1/ACT1::* P_{ACT1}*-GFP-ATG8*	This study
WT+GFP-*ATG1*	*ura3Δ::λimm434/ura3Δ::λimm434*<br>*his1::hisG/his1::hisG arg4::hisG/arg4::hisG*<br>*ACT1/ACT1::* P_{ACT1}*-GFP-ATG1*	Jiawen Du
mec1Δ/Δ-GFP-*ATG1*	*ura3Δ::λimm434/ura3Δ::λimm434*<br>*his1::hisG/his1::hisG arg4::hisG/arg4::hisG*<br>*mec1::ARG4/mec1:: URA3-dpl200*<br>*ACT1/ACT1::* P_{ACT1}*-GFP-ATG1*	This study
rad53Δ/Δ-GFP-*ATG1*	*ura3Δ::λimm434/ura3Δ::λimm434*<br>*his1::hisG/his1::hisG arg4::hisG/arg4::hisG*<br>*rad53::ARG4/rad53:: URA3-dpl200*<br>*ACT1/ACT1::* P_{ACT1}*-GFP-ATG1*	This study
WT+GFP-*ATG13*	*ura3Δ::λimm434/ura3Δ::λimm434*<br>*his1::hisG/his1::hisG arg4::hisG/arg4::hisG*<br>*ACT1/ACT1::* P_{ACT1}*-GFP-ATG13*	Jiawen Du
mec1Δ/Δ-GFP-*ATG13*	*ura3Δ::λimm434/ura3Δ::λimm434*<br>*his1::hisG/his1::hisG arg4::hisG/arg4::hisG*<br>*mec1::ARG4/mec1:: URA3-dpl200*	This study

	ACT1/ACT1:: P_{ACT1}*-GFP-ATG13*	
*rad53*Δ/Δ-GFP-*ATG13*	*ura3Δ::λimm434/ura3Δ::λimm434*	This study
	his1::hisG/his1::hisG arg4::hisG/arg4::hisG	
	rad53::ARG4/rad53:: URA3-dpl200	
	ACT1/ACT1:: P_{ACT1}*-GFP-ATG13*	
mec1Δ/Δ	*ura3Δ::λimm434/ura3Δ::λimm434*	This study
	his1::hisG/his1::hisG arg4::hisG/arg4::hisG	
	mec1::ARG4/mec1:: URA3-dpl200	
Rad53Δ/Δ	*ura3Δ::λimm434/ura3Δ::λimm434*	This study
	his1::hisG/his1::hisG arg4::hisG/arg4::hisG	
	rad53::ARG4/rad53:: URA3-dpl200	
WT-*CSP37*-GFP	*ura3Δ::λimm434/ura3Δ::λimm434*	Qilin Yu
	his1::hisG/his1::hisG arg4::hisG/arg4::hisG	
	CSP37/CSP37:: CSP37-GFP	
WT-*SEC61*-GFP	*ura3Δ::λimm434/ura3Δ::λimm434*	Qilin Yu
	his1::hisG/his1::hisG arg4::hisG/arg4::hisG	
	SEC61/SEC61:: SEC61-GFP	
*psp2*Δ/Δ-GFP-*ATG8*	*ura3Δ::λimm434/ura3Δ::λimm434*	This study
	his1::hisG/his1::hisG arg4::hisG/arg4::hisG	
	psp2::ARG4/psp2:: URA3-dpl200	
	ACT1/ACT1:: P_{ACT1}*-GFP-ATG8*	
PSP2c-GFP-*ATG8*	*ura3Δ::λimm434/ura3Δ::λimm434*	This study

	his1::hisG/his1::hisG arg4::hisG/arg4::hisG	
	psp2::ARG4/psp2:: URA3-dpl200	
	his1::hisG/his1:: P_{PSP2}*-PSP2*	
	ACT1/ACT1:: P_{ACT1}*-GFP-ATG8*	
dcp2Δ/Δ-GFP-*ATG8*	*ura3Δ::λimm434/ura3Δ::λimm434*	This study
	his1::hisG/his1::hisG arg4::hisG/arg4::hisG	
	dcp2::ARG4/dcp2:: URA3-dpl200	
	ACT1/ACT1:: P_{ACT1}*-GFP-ATG8*	
DCP2c-GFP-*ATG8*	*ura3Δ::λimm434/ura3Δ::λimm434*	This study
	his1::hisG/his1::hisG arg4::hisG/arg4::hisG	
	dcp2::ARG4/dcp2:: URA3-dpl200	
	his1::hisG/his1:: P_{DCP2}*-DCP2*	
	ACT1/ACT1:: P_{ACT1}*-GFP-ATG8*	
WT-*ATG1*-HA	*ura3Δ::λimm434/ura3Δ::λimm434*	This study
	his1::hisG/his1::hisG arg4::hisG/arg4::hisG	
	ATG1/ATG1::ATG1-HA	
psp2Δ/Δ-*ATG1*-HA	*ura3Δ::λimm434/ura3Δ::λimm434*	This study
	his1::hisG/his1::hisG arg4::hisG/arg4::hisG	
	psp2::ARG4/psp2:: URA3-dpl200	
	ATG1/ATG1::ATG1-HA	
dcp2Δ/Δ-*ATG1*-HA	*ura3Δ::λimm434/ura3Δ::λimm434*	This study
	his1::hisG/his1::hisG arg4::hisG/arg4::hisG	

	dcp2::ARG4/dcp2:: URA3-dpl200	
	ATG1/ATG1::ATG1-HA	
WT-*ATG13*-HA	*ura3Δ::λimm434/ura3Δ::λimm434*	This study
	his1::hisG/his1::hisG arg4::hisG/arg4::hisG	
	ATG13/ATG13::ATG13-HA	
*psp2*Δ/Δ-*ATG13*-HA	*ura3Δ::λimm434/ura3Δ::λimm434*	This study
	his1::hisG/his1::hisG arg4::hisG/arg4::hisG	
	psp2::ARG4/psp2:: URA3-dpl200	
	ATG13/ATG13::ATG13-HA	
*dcp2*Δ/Δ-*ATG13*-HA	*ura3Δ::λimm434/ura3Δ::λimm434*	This study
	his1::hisG/his1::hisG arg4::hisG/arg4::hisG	
	dcp2::ARG4/dcp2:: URA3-dpl200	
	ATG13/ATG13::ATG13-HA	
WT-*MEC1*-HA	*ura3Δ::λimm434/ura3Δ::λimm434*	This study
	his1::hisG/his1::hisG arg4::hisG/arg4::hisG	
	MEC1/MEC1::MEC1-HA	
*psp2*Δ/Δ-*MEC1*-HA	*ura3Δ::λimm434/ura3Δ::λimm434*	This study
	his1::hisG/his1::hisG arg4::hisG/arg4::hisG	
	psp2::ARG4/psp2:: URA3-dpl200	
	MEC1/ MEC1:: MEC1-HA	
*dcp2*Δ/Δ-*MEC1*-HA	*ura3Δ::λimm434/ura3Δ::λimm434*	This study
	his1::hisG/his1::hisG arg4::hisG/arg4::hisG	

	dcp2::ARG4/dcp2:: URA3-dpl200 *MEC1/MEC1::MEC1-HA*	
WT-*RAD53*-HA	*ura3Δ::λimm434/ura3Δ::λimm434* *his1::hisG/his1::hisG arg4::hisG/arg4::hisG* *RAD53/RAD53::RAD53-HA*	This study
*psp2*Δ/Δ-*RAD53*-HA	*ura3Δ::λimm434/ura3Δ::λimm434* *his1::hisG/his1::hisG arg4::hisG/arg4::hisG* *psp2::ARG4/psp2:: URA3-dpl200* *RAD53/ RAD53:: RAD53-HA*	This study
*dcp2*Δ/Δ-*RAD53*-HA	*ura3Δ::λimm434/ura3Δ::λimm434* *his1::hisG/his1::hisG arg4::hisG/arg4::hisG* *dcp2::ARG4/dcp2:: URA3-dpl200* *RAD53/RAD53::RAD53-HA*	This study
WT (URA$^+$)	*URA3/ura3Δ::λimm434/ura3Δ::λimm434* *his1::hisG/his1::hisG arg4::hisG/arg4::hisG*	Qilin Yu
*mec1*Δ/Δ (URA$^+$)	*URA3/ura3Δ::λimm434/ura3Δ::λimm434* *his1::hisG/his1::hisG arg4::hisG/arg4::hisG* *mec1::ARG4/mec1::dpl200*	This study
*rad53*Δ/Δ (URA$^+$)	*URA3/ura3Δ::λimm434/ura3Δ::λimm434* *his1::hisG/his1::hisG arg4::hisG/arg4::hisG* *dcp2::ARG4/dcp2::dpl200*	
**Plasmids**		
pP$_{ACT1}$-GFP-Atg8	Amp^r, containing *URA3* marker	Qilin Yu

pP$_{ATG13}$-*ATG13*	Containing *HIS3* marker, Amp^r	This study
pP$_{ATG13}$-*ATG13*$^{1\text{-}268aa\Delta/\Delta}$	Containing *HIS3* marker, Amp^r	This study
pP$_{ATG13}$-*ATG13*$^{269\text{-}738aa\Delta/\Delta}$	Containing *HIS3* marker, Amp^r	This study
pP$_{ATG13}$-*ATG13*$^{461\text{-}640aa\Delta/\Delta}$	Containing *HIS3* marker, Amp^r	This study
pP$_{ATG13}$-*ATG13*$^{461\text{-}474aa\Delta/\Delta}$	Containing *HIS3* marker, Amp^r	This study
pP$_{ACT1}$-GFP-Atg1	Amp^r, containing *URA3* marker	Jiawen Du
pP$_{ACT1}$-GFP-Atg13	Amp^r, containing *URA3* marker	This study
pRS-ArgΔSpe I	Containing *ARG4* marker, Amp^r	Dana Davis
pDDB57	Containing *URA3* marker, Amp^r	Dana Davis
pDDB78	Containing *HIS3* marker, Amp^r	Dana Davis
Pp$_{MEC1}$-*MEC1*	Containing *HIS3* marker, Amp^r	This study
Pp$_{RAD53}$-*RAD53*	Containing *HIS3* marker, Amp^r	This study
Pp$_{PSP2}$-*PSP2*	Containing *HIS3* marker, Amp^r	This study
Pp$_{DCP2}$-*DCP2*	Containing *HIS3* marker, Amp^r	This study
pGFP-URA3	Amp^r, containing *URA3* marker	Qilin Yu
pHA-URA3	Amp^r, containing *URA3* marker	Qilin Yu
pLUBP	Containing a 4.9 kb *IRO1-URA3* cassette	Gerald Fink
