# Supplementary material for: Mec1-Rad53 Signaling Regulates DNA Damage-Induced Autophagy and Pathogenicity in Candida albicans
Source: J Fungi (Basel). 2023 Dec 9;9(12):1181. doi: 10.3390/jof9121181 (PMC10744610; doi:10.3390/jof9121181)
Supplement: Supplementary file 1 [file jof-09-01181-s001.zip › TableS2.pdf]

TableS2 Primers used in this study.

Primers	Sequence (5′-3′)
ATG13(WT)-1F	TCCCCCGGGGGATTCCCCACCACGTCTTTTATC
ATG13(WT)-1R	GGGGTACCCCTTATTCGTTTTGGGGCTGGTTG
ATG13(269-738aaΔ/Δ)-1R	GATTAAAGCTCTTTTTCTATTTTCATTCATGTAGACAA AACTCGGAATCC
ATG13(269-738aaΔ/Δ)-2F	GGATTCCGAGTTTTGTCTACATGAATGAAAATAGAAA AAGAGCTTTAATC
ATG13(1-268aaΔ/Δ)-1R	GAAATGGGACAGTAGTAACTCTTCATTAGTTAGAAGA ATAGGTATCGTATTC
ATG13(1-268aaΔ/Δ)-2F	GAATACGATACCTATTCTTCTAACTAATGAAGAGTTAC TACTGTCCCATTTC
ATG13(461-640aaΔ/Δ)-1R	GTTACATGAGGTATTCTTGAACTTATGGTTTGACCCAT TAAATCGGATTGG
ATG13(461-640aaΔ/Δ)-2F	CCAATCCGATTTAATGGGTCAAACCATAAGTTCAAGA ATACCTCATGTAAC
ATG13(461-474aaΔ/Δ)-1R	CATACGGACAAAGTCACTTATGTCGGTTTGACCCATT AAATCGGATTGG
ATG13(461-474aaΔ/Δ)-2F	CCAATCCGATTTAATGGGTCAAACCGACATAAGTGAC TTTGTCCGTATG
MEC1-KO-F	CATTTCTTATTTGTATATTATTAAATACGTATATCGCGA GAAACTGTATAAATACTCTTGTTTCCCAGTCACGACG TT
MEC1-KO-R	CATCTGGGTTACTCATTAATTGGTATGTATACTGGACT ATGTTCAACGTCTTACGAATAACGTGGAATTGTGAGC GGATA
MEC1-DE-F	TCAAGAGACCCTTAAATCC
MEC1-DE-R	CATCTGTATAGTTTAATGA
MEC1-IN-F	ATGACGTCGAATCAATCAAT

MEC1-IN-R	CCAAATTTTCCTACTACAACTC
MEC1-CP-F	CTAGCTAGCTAGATGACGTCGAATCAATCAAT
MEC1-CP-R	TCCCCCGGGGGACATATAAGCTGCCCAACCTG
RAD53-KO-F	GTTAATGTTGTATGTTGAAGATTTGATGTTGAAAAAGAAAAAAGATTTGTTCAAAATTGTTTCCCAGTCACGACGTT
RAD53-KO-R	GTAAATGAGAAGTTAAAAGTACATAAACCAAACATCCCAAAGACCAAATGTCAACCAAGGGTGGAATTGTGAGCGGATA
RAD53-DE-F	GTTTGTAGATTGCCTGACG
RAD53-DE-R	GATTGCGATAAACTCAATG
RAD53-IN-F	ATGGAAGTAACACAACGGAC
RAD53-IN-R	TGCGTCTTCTCCTATTGCAC
RAD53-CP-F	ATTTGCGGCCGCTTTAGTAACCACATCAACATTGG
RAD53-CP-R	CCATCGATGGGTTTAGTATCTCGGTATATAC
ATG1-NG-F	TCCCCCGGGGGAATGATACCTCAGTCGAACC
ATG1-NG-R	CGGGATCCCGTCATTTCCCCATGAAATTC
ATG13-NG-F	TCCCCCGGGGGAATGTTGTCAGATTTCAAAC
ATG13-NG-R	CGGGATCCCGTCAAAAATTCCTTGAATTC
PSP2-KO-F	GATTAATTAATCTACTGCTTTTTAATTGATTGACTTTAATTACTAACAGAATTATATTTTTTTCCCAGTCACGACGTT
PSP2-KO-R	CAACTGGTTTGGCACCACCAAATGGATTTGGCTTGGGTTTAGAAGTGGAACTGGCAGATGAGTGGAATTGTGAGCGGATA
PSP2-DE-F	GTATTTTAATTTAATTTATTC
PSP2-DE-R	CTTCGTCGGGGTTTTCTTC
PSP2-IN-F	ATGTCCTTACAGGAGTTTTTC
PSP2-IN-R	CTTTCAAAGTCGTCTAAAAG

PSP2-CP-F	ATTTGCGGCCGCTTTAGATGTTACGGTGATTATAT
PSP2-CP-R	CCATCGATGGGTGAGAAATCAGTGTTACAG
DCP2-KO-F	CACACAAGTTGTTTATCCAGTATACAAACATCAATACAATTTTGAATCAATTTTAATTACTTTCCCAGTCACGACGTT
DCP2-KO-R	GTTAGTCTTGGGCTTCTGAAAGCTTTGAGGGTTAGGGGGTTGTTGGTTATGTGGACTAACATGTGGAATTGTGAGCGGATA
DCP2-DE-F	CGTTTACCAGTCCACAAGG
DCP2-DE-R	CAAGTTCATTCTCATTCTCAAC
DCP2-IN-F	ATTTGTTAGTTAGATTTGTG
DCP2-IN-R	CATAATTTCTTCCTCGTTC
DCP2-CP-F	ATTTGCGGCCGCTTTAGCTTAATCTATTGTCTAATG
DCP2-CP-R	CCATCGATGGGAGTCTATTTGCTCTAGG
ATG1-HA-F	CAGGAAGTGAAGAAAATAATTGAAAAAATTAACTCCCGTTTGAAGAATTTCATGGGGAAACCCGGGTACCCATACGATGTTC
ATG1-HA-R	CGAAGAGACAAAGGATAAGGTAGCAGAAATTAGTGAAAAGGTTGAAGATGTCAAGGAAGCTAGAAGGACCACCTTTG
ATG1-IN-F	GTAAAAGTGATTTTACTGCTG
ATG13-HA-F	GATAGTGAAGATGACGAAGATTTGTTGTTTACTATGAGTGATATGAATTCAAGGAATTTTCCCGGGTACCCATACGATGTTC
ATG13-HA-R	CTATGACTTGCATCAGGTGAAAACAGGAATGACGGCTTTCAAATTTGGGAGGAAACTTGCCTAGAAGGACCACCTTTG
ATG13-IN-F	AACAAACTCCCCAATCCGAT
URA3-IN-F	CCTATAGTGAGAGAGCAG

URA3-IN-R	ATGGAAGTAACACAACGGAC
MEC1-HA-F	CAAGAAGCTACATCATTAGAGAGATTATCACAAATGTATGCAGGTTGGGCAGCTTATATGCCCGGGTACCCATACGATGTTC
MEC1-HA-R	ATAAAAGATTTATAGTACATAATAAGAAGTAGATTTGTTATGAAATTTGACATTTATTCACTAGAAGGACCACCTTTG
MEC1-IN-F	GTCCGCCTGTTTTACATACT
RAD53-HA-F	CAAACCTCAGAAGAAATGAATATTATTCCACTTATTTCGGGTTTAAGTAGTATAAGTTCACCCGGGTACCCATACGATGTTC
RAD53-HA-R	GTTCGGTTGCTATCTCAATCATTGCGTTTAGTATCTCGGTATATACCCCCACAGTTTTATCCTAGAAGGACCACCTTTG
RAD53-IN-F	ATGGAAGTAACACAACGGAC
